# Assessing adolescent diet and physical activity behaviour, knowledge and awareness in low- and middle-income countries: a systematised review of quantitative epidemiological tools

**DOI:** 10.1186/s12889-022-13160-6

**Published:** 2022-05-14

**Authors:** Trish Muzenda, Monika Kamkuemah, Jane Battersby, Tolu Oni

**Affiliations:** 1 Global Diet and Physical Activity Research, MRC Epidemiology Unit, University of Cambridge, Cambridge, CB2 0QQ UK; 2 Research Initiative for Cities Health and Equity (RICHE), Division of Public Health Medicine, School of Public Health and Family Medicine, University of Cape Town, Cape Town, 7925 South Africa; 3 African Centre for Cities, University of Cape Town, Cape Town, 7945 South Africa

**Keywords:** Adolescent, Diet, Physical activity, Assessment, Epidemiological tools, low- and middle-income countries, LMIC

## Abstract

**Purpose:**

Quantitative epidemiological tools are routinely used to assess adolescent diet and physical activity (PA) constructs (behaviour, knowledge, and awareness) as risk factors for non-communicable diseases. This study sought to synthesize evidence on the quantitative epidemiological tools that have been used to assess adolescent diet and PA constructs in low to middle-income countries (LMIC).

**Methods:**

A systematised review was conducted using 3 databases (EbscoHost, Scopus and Web of Science).

**Results:**

We identified 292 LMIC studies assessing adolescent diet and PA. Identified studies predominantly explored behavioural (90%) constructs with a paucity of studies investigating knowledge and awareness. The majority of studies used subjective (94%) and self-administered (78%) tools. Only 39% of LMIC studies used tools validated for their contexts.

**Conclusions:**

The findings highlight the need for more contextual tools for assessing adolescent diet and PA in LMICs. Diet and PA measurement tools used in future research will need to incorporate measures of knowledge and awareness for a more comprehensive understanding of the epidemiology of diet and PA in adolescents. Furthermore, there is a need for more evidence on the reliability and validity of these tools for use, in both cross sectional and longitudinal studies, in LMIC contexts.

**Supplementary Information:**

The online version contains supplementary material available at 10.1186/s12889-022-13160-6.

## Background

The burden of non-communicable disease (NCD) is disproportionately higher in low- and middle-income countries (LMICs) [1]. In 2019, 75% of global all-cause mortality was attributable to NCDs, with 77% of NCD deaths reported in LMICs [1, 2]. Increasing morbidity and mortality, particularly in LMICs, has prompted the need for innovative interventions to address modifiable NCD risk factors, notably unhealthy diet and physical inactivity [3]. Diet and physical activity (PA) are both implicated in the aetiology of the main NCDs-cardiovascular diseases, cancers, respiratory diseases, and diabetes [3, 4]. In LMICs, NCD occurrence is disproportionally higher in adults aged above 30 years but associated behavioural risk factors begin to develop and are reinforced during adolescence. The life course approach recognises that an individual’s health status is a function of past (and present) biopsychosocial pathways operating at key developmental stages [5]. This approach further recognizes that developmental and behavioural changes [6, 7] occurring at the adolescent stage provide opportunities for disease prevention through the promotion of healthy diets and PA.

The development and implementation of contextually appropriate NCD prevention strategies in LMICs relies on the ability to accurately collect data on adolescent diet and PA behaviours. At the global level, various epidemiological tools have been developed to provide quantitative and qualitative measures of diet and PA; with these tools being either objective or subjective [8, 9]. Subjective tools rely on individual self-report of diet or PA constructs over defined periods. Examples include questionnaires and logbooks [10, 11]. Conversely, objective tools quantify dietary or PA constructs by recording phenomena that translate to dietary or PA estimates [12]. Examples include accelerometers that measure body movement to estimate PA [12, 13], and nutritional biomarkers that assess dietary consumption [8].

Research evidence indicates an increasing number of studies exploring adolescent diet and PA trends in LMICs. However, the majority of tools used by these studies to assess adolescent diet and PA behaviours and knowledge have been developed in high-income country (HIC) contexts [14] which may not adequately capture adolescent diet and PA behaviours in LMIC contexts.

This systematised literature review sought to identify and describe the quantitative epidemiological tools used for assessing adolescent diet and physical activity in LMICs, using the constructs of behaviour, knowledge, and awareness. We further explored the origin and validation of these tools for use in LMICs.

## Methods

### Overview

The systemized literature review typology was selected to guide our literature search. Systematised reviews combine elements from both systematic and traditional literature reviews [15], and follow a comprehensive search strategy that is uniformly executed across databases to identify relevant literature. Quality assessment of retrieved articles is not required [15].

### Search strategy

**Fig. 1:**
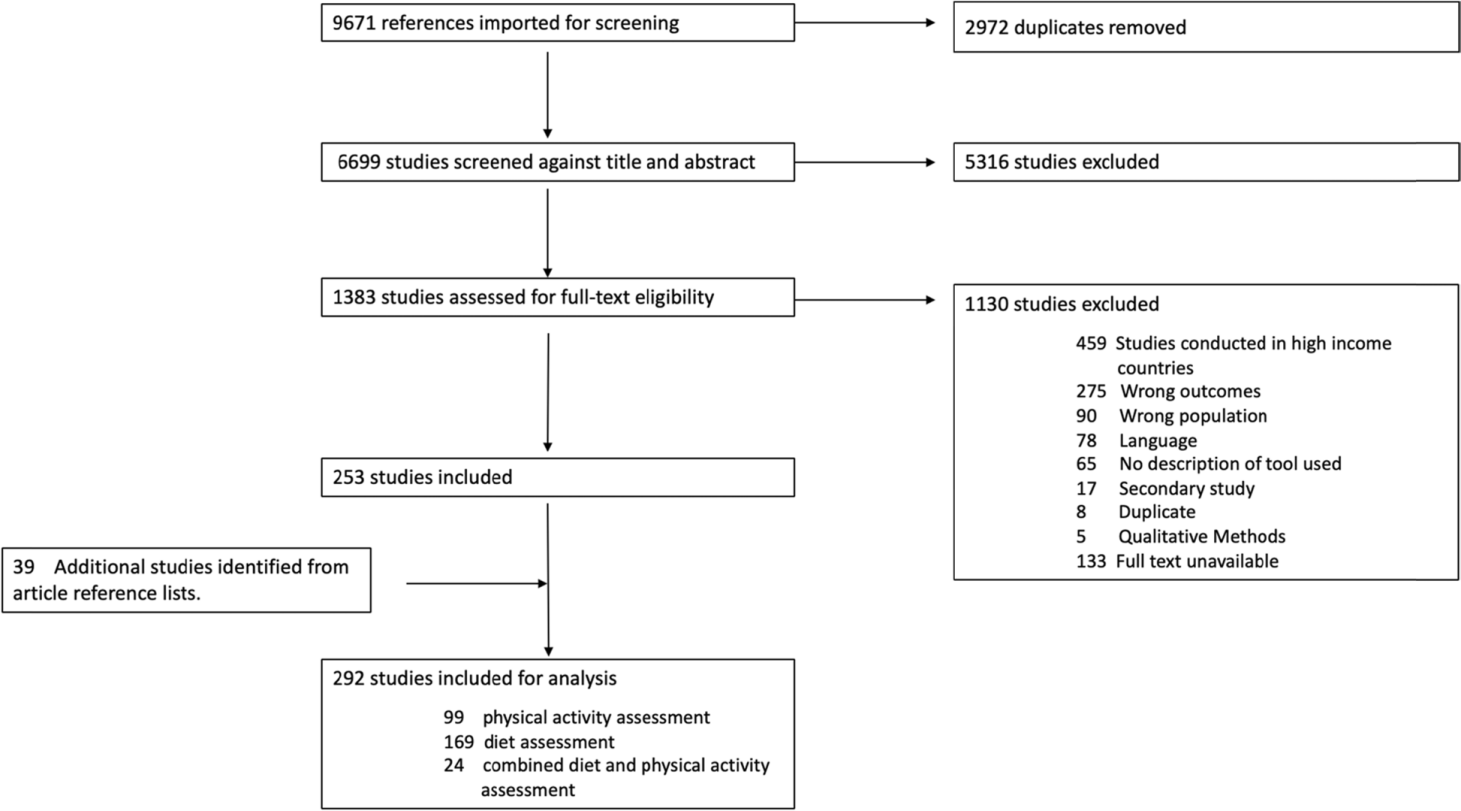
PRISMA Diagram of the literature review process

An initial search was conducted in Google Scholar and PubMed to identify key literature and keywords for use in the literature search. The finalised literature search strategy (Additional file 1 Tables 1A and 1B) included variations of the following terms “knowledge”, “awareness”, “behaviour” “epidemiology”, “adolescents”, “physical activity” and “diet”. Between August – December 2019, the search terms were applied uniformly across three databases – EbscoHost [16], Scopus [17] and Web of Science [18]. The search yielded a total of 9098 studies (Scopus – 3688, EbscoHost – 3845, Web of Science- 1565). Article abstracts were uploaded onto Covidence [19], a web-based online screening software, for both abstract and full text screening. Duplicates (*n* = 2922) were automatically removed by Covidence. Titles and abstracts (*n* = 6176) were screened by two independent reviewers (authors TM and MK) to identify eligible studies that met the inclusion criteria. Full text screening of the remaining 1337 articles was undertaken by the same two reviewers. Any discrepancies were resolved by a third reviewer. The systematised search was subsequently updated in January 2022. A total of 573 (Scopus – 106, EbscoHost – 215, Web of Science – 252) studies were identified. Retrieved studies underwent title, abstract and full text screening, of which 26 studies met the inclusion criteria. The reference lists of included studies were thereafter examined to identify additional relevant studies. This process yielded a further 39 articles. As highlighted in the PRISMA flow diagram (Fig. 1), 292 LMIC studies were included for data analysis.

### Inclusion criteria

To meet the research objective, the following inclusion criteria were applied: the main outcome included reported scores for adolescent diet or PA; the study utilised quantitative epidemiological approaches in both data collection and analysis; study population included adolescents aged between 10 and 19 years; articles were written in English or translated versions were available. There were no limitations on geographical location. However, given the focus on studies from LMICs, locations were recorded to highlight the country in which the studies were conducted. The World Bank Group’s income classification grouping was used to identify LMICs (Additional file 2) [20]. To capture instrument diversity, there were no limitations on the date of publication or on data collection methods. Studies were excluded from the literature review if they did not meet these criteria (Fig. 1).

## Results

We identified 292 studies conducted in LMICs. Of these, 24 assessed diet and PA in the same study (Additional file 3 Table 3A), 99 studies assessed PA constructs only (97% assessed behaviour; 3% knowledge and awareness) (Additional file 3 Table 3B), and 169 studies assessed dietary constructs only (86% assessed behaviour; 14% knowledge and awareness) (Additional file 3 Table 3C).

### Assessing adolescent physical activity constructs

The majority (97%) of studies assessed PA behaviour (both subjectively and objectively) (*n* = 96/99). The remaining three studies assessed PA knowledge and awareness exclusively (*n* = 1) or concurrently with PA behaviour (*n* = 2). PA behaviour was reported under four domains – transport, household, occupational (school related) and leisure time. Leisure time PA encompassed walking, running, recreational sport and playful activities.

#### Physical activity behaviour

LMIC adolescent PA behaviour was predominantly measured using subjective tools – exclusive questionnaires (*n* = 75), questionnaires in conjunction with another tool (n = 7), 24-h PA recall reports (*n* = 3), and one PA real-time record. (Additional file 3 Table 3B). Utilised questionnaires included tools originally developed in HICs (*n* = 47), regionally (*n* = 4) and locally (*n* = 25). Tools developed in HIC included the International Physical Activity Questionnaire (IPAQ) (*n* = 15) [21–35], Physical Activity Questionnaire (PAQ) (*n* = 5) [21, 36–39], Health Behavior School-aged Children Survey Questionnaire (HBSC) [40] and the Three Day Physical Activity Recall (3DPAR) [41].

We found two regional tools, Arab Teens Lifestyle Study (ATLS) [42–47] and South American Youth/Child Cardiovascular and Environment (SAYCARE) [48], developed for use in Arab and Latin American countries, respectively. The development of regional tools was guided by pre-existing tools with adaptations made to suit the regional context. For example, the SAYCARE questionnaire assessed 3 domains of PA - school, leisure time and transport - as adolescents living in the region were most likely to engage in moderate to vigorous PA [48]. Similarly, in adapting to the cultural context in the Middle-East region, only girls were asked about their participation in dancing as a means for PA in the ATLS questionnaire [42–44, 49].

Nineteen studies used locally developed (country-specific) tools. These studies were conducted in 11 countries – Brazil (*n* = 6) [50–55], Malaysia (*n* = 3) [56–58], Thailand (*n* = 1) [59], Vietnam (*n* = 2) [60, 61], Mozambique (*n* = 2) [62, 63], India (*n* = 1) [64], China (*n* = 1) [65], Colombia (*n* = 1) [66], Ecuador (*n* = 1) [67], Iran (*n* = 1) [68], and Ghana (*n* = 1) [69]. Although developed locally, the questions and PA domains were similar to those used in HICs.

Only 16 studies used objective measures to assess LMIC adolescent PA using accelerometery (*n* = 14), heart rate monitors (*n* = 1), pedometer (*n* = 1) and direct observation (*n* = 1). We also found 5 studies that utilised a combination of questionnaires and accelerometers concurrently when assessing PA [35, 70–73].

#### Physical activity knowledge and awareness

We identified three LMIC studies assessing PA knowledge and awareness using bespoke questions [74–76]. These studies assessed perceived benefits and barriers to regular PA [74], PA self-efficacy [76], and the association between health and PA [75].

### Assessing adolescent dietary constructs

We identified 169 studies (Additional file 3 Table 3C) assessing adolescent dietary constructs only, with a majority of the studies (*n* = 145) assessing behaviour. Behaviour constructs included the quantity, frequency and type of food habitually consumed. Adolescent dietary knowledge and awareness were assessed exclusively in seven studies, with an additional 17 studies measuring knowledge and awareness alongside dietary behaviour.

#### Dietary behaviour

Of the 145 studies documenting adolescent dietary behaviour, 108 used questionnaires, 11 dietary records, 17 dietary recall, one used an observational method and nine used a combination of questionnaires and dietary recall methods.

Internationally developed tools were used in 27 LMIC studies. Examples of tools used include the Global School Health Survey – GSHS [77, 78], WHO & FAO 1 day diversity questionnaire [79], and the Nutrition Transition Food Frequency Questionnaire - NT-FFQ [80]. The Arabic Eating Habits Questionnaire (AEHQ) was the only identified LMIC regional tools assessing diet [81]. The AEHQ aimed to identify the food consumption and snacking patterns of Arab adolescents and included a food list characteristic of the Arab diet as well as a dedicated segment to assess fast food consumption.

The majority of questionnaires (*n* = 71) used were either adapted (from regional or international tools) or specifically developed (country-specific) for use in the country. For example the Adolescent Eating Attitudes Questionnaire (Brazil) was adapted from the Project EAT (Eating Among Teens) questionnaire (USA) [82], and a food frequency questionnaire (FFQ) (Jamaica) was adapted from a regional 1997 Caribbean Youth Survey [83]. Other country-specific questionnaires used bespoke food frequency questionnaires (FFQs) that contained contextually relevant food lists designed to capture the variation in local diets. Consequentially, although country-specific tools all assessed dietary behaviour, differences in the actual content limited the comparability of study findings across different populations.

Eleven studies reported dietary behaviour using prospective dietary records over periods ranging from 24 h, 1 week to 3 months [84–94]. Participants were first trained on data collection procedures and thereafter kept detailed records (in a notebook) of dietary practices over a set period, typically 24 h. In some instances, dietary records allowed researchers to collect information on food preparation methods as exemplified in a Brazilian study where participants took notes on food preparation methods as well as places of consumption [86]. Paper notebooks were the main equipment for recording data.

The dietary recall method was used in 17 studies by asking adolescents to recall all the food they had consumed in the previous day [95–111]. Using the dietary recall method, participants provided information on food consumed in the preceding 24 h over one to 7 days depending on the objectives of the study. In seven of these studies, participant recall was facilitated by a researcher providing questions and prompts to aid recall [95, 96, 99, 100, 106, 107, 112]. The 24-h time period used in dietary recall assessments gave an indication of food intake. However, there was potential to overlook infrequently consumed foods that contribute towards an individual’s total food intake. To mitigate this limitation, some studies conducted dietary recall every 24 h over two to 7 days [96, 103, 113].

#### Dietary knowledge and awareness

We identified 24 questionnaires investigating adolescent dietary knowledge and awareness. Adolescent nutritional knowledge was assessed in 13 studies using close-ended questions [104, 113–124]. Nine studies used questionnaires to assess information on dietary attitudes, perceptions, and food choices [92, 104, 114, 116, 125–129]. Examples include a Turkish study that measured knowledge of a local Food Guide Pyramid (FGP), by asking adolescents to list food groups recommended in the FGP in order of recommended consumption frequency relative to their own daily consumption [130].

### Validation and reliability of tools used in low- and middle-income countries

Only 39% (*n* = 113/292) of the 292 studies used subjective epidemiological tools validated for use in their geographical contexts. Measures of reliability included test-retest reliability (*n* = 51) and internal consistency (*n* = 17). Validation methods were construct validity (*n* = 4), content validity (*n* = 24), criterion validity (*n* = 43) and face validity (*n* = 17), with some studies utilising multiple methods. The types of validation methods used are detailed in Additional file 3 (Tables 3A, 3B, 3C). Tool validation was described either as part of the study methods or in reference to prior studies showing validity and reliability of the tool in a similar geographical context. With respect to validation of tools for the adolescent population group, 58% (*n* = 65/113) of studies using validated tools specifically mentioned use of tools originally designed for adolescents. None of the identified objective tools, were explicitly validated for use in LMIC settings.

#### Validation of tools measuring PA constructs

Of the 113 studies detailing validation methods in LMIC, 50 were focused on PA (46 questionnaires, 1 PA recall, 1 pedometer and 2 logbook). When disaggregated by tool origin, 29 studies used tools initially developed in HICs, 4 studies used regionally developed tools and 16 were developed within the country. Validated tools developed in HIC included the IPAQ (*n* = 13), PAQ (*n* = 4), 3DPAR (*n* = 1) and the GSHS (*n* = 1), with validation conducted to assess suitability of tools to the local contexts. Face validity of the IPAQ was consistently assessed in different regions including Brazil, Iran, Thailand, Turkey and Macedonia to assess if participants and subject experts thought, at face value, these tools where appropriate for assessing PA in the local context [27, 28, 31, 33–35, 131–135]. The content validity of international tools (IPAQ, GSHS and PAQ) was assessed in 10 studies [22, 27, 33, 35, 39, 60, 133, 136–138]. For example, after translation, the GSHS was validated by a group of experts prior to use in Iran [33]. Construct validity of international tools was also assessed in Indonesia [28], Brazil [139], and Ecuador [67].

In addition to international tools, we found two regional tools that had been validated for local contexts – ATLS [42–44] and the SAYCARE [48]. Although ATLS was initially comparatively validated against pedometer readings in adolescents, subsequent studies further tested the tool for reproducibility (using the test-retest method) [43]. The SAYCARE was tested for reproducibility and criterion validity (against accelerometery) in seven cities (Lima, Buenos Aires, Medellin, Montevideo, Santiago, Sao Paulo and Teresina) [48].

Four questionnaires identified were developed and validated at the country level: Thailand PA Children Survey (TPACS-SQ), Vietnamese Adolescent PA Recall Questionnaire (VAPARQ), Madras Diabetes Research Foundation – Physical Activity Questionnaire for Children and Adolescents (MPAQ©) and, National Adolescent School-based Health Survey (Brazil (PeNSE) [53, 59, 61, 64]. The TPACS-SQ was developed by modifying a previous questionnaire, Child and Adolescent PA and Nutrition Survey (CAPANS) which was validated in comparison to accelerometery [59]. Given that the contents and constructs of the CAPANS had demonstrated validity, the current TPACS-SQ was tested for reproducibility using the test-retest method. The VAPARQ was adapted and translated from the Australia Adolescent Physical Activity Recall Questionnaire (APARQ) and given the tropical climate in Vietnam, validation involved qualitative discussions to identify the range of activities to be included [61].

#### Validation of tools measuring dietary constructs

Fifty-three studies highlighted the use of validated tools in the assessment of adolescent dietary constructs (50 questionnaires, one dietary record and two dietary recall). Of these, 16 were developed in HICs. These tools included the GSHS (Iran and Uganda) [77, 78], Eating Habits Questionnaire for Adolescents – (Ghana) [140], Adolescent Food Frequency Questionnaire (Brazil) [141], Block Questionnaire (Brazil) [142], Food Consumption Markers form (Brazil) [143]. To test reproducibility, eight studies used the test-retest methods [77, 78, 80, 141, 144–147], and six used the internal consistency method in pilot studies [119, 140, 144, 148–150]. Content validity was assessed for the GSHS in Iran [78], NTFFQ in Brazil [151], and Adolescents’ Knowledge of Healthy Eating Questionnaire in Nigeria [148] to ensure that the tool assessed all facets of dietary consumption in adolescents.

Twenty-eight studies developed and validated context specific FFQs within their country. Examples include an FFQ to assess adolescent food consumption amongst Isfahan female students that consisted of a 53-item food list of items commonly consumed in Iran [152]. Similarly, a Brazilian study used a food list with food items commonly eaten by adolescents based of previous 24 h food records [153].

Of the 50 validated dietary questionnaires identified, 14 used criterion validity against the 24-h dietary recall method [104, 115, 154–170]. Validity was confirmed if there was correlation between dietary scores or outcomes from both methods. Face validity was estimated in six studies by asking experts in nutrition, behaviour and education to assess the suitability of questions presented in the questionnaire [78, 127, 144, 148, 150, 171]. Content validity was conducted in a similar fashion [146, 148, 151, 171–174]. Reproducibility of tools was assessed using the test-retest and internal consistency methods.

#### Validation of tools measuring both dietary and PA constructs

We identified 10 questionnaires assessing both food and PA concurrently that were also validated for their contexts [49, 175–183]. Tools used included the Global School Based Student Health Survey (GSHS) [176, 178], ATLS [49], and a combination of diet (FFQ) and PA (IPAQ, PAQ-A tools) [175, 177, 181]. Validation was assessed using a combination of face, content, and construct, and criterion validation methods.

### Tool administration

The majority of studies used school-based adolescent sampling strategies (75%, *n* = 218/292). Two types of tool administration were utilised in LMIC studies: self- (*n* = 230/292, 79%) and researcher-led (*n* = 64, 21%) administration. For both types, questions were first explained to the adolescents at the beginning or during data collection with opportunities to ask questions made available by the researcher. Other methods such as dietary and PA recall and records lend themselves to self-administration by design.

When disaggregated, 10% (*n* = 10/99), 28% (*n* = 48/169), and 25% (*n* = 6/24) of studies assessing PA, diet, and combined PA and diet, respectively, used interviewer-administered tools for data collection. Interview administration for dietary assessment was reported to be useful in probing the participant to report in detail their dietary habits or awareness. However, studies acknowledged limitations associated with interviewer administration, noting efforts to reduce bias when possible.

Objective assessments of PA (*n* = 17) were self-administered (with guidance from research team members), because the devices used in data collection -accelerometers, pedometers, and heart rate monitors – are either attached or worn by the adolescent over a set period of data collection.

Nearly half of the studies used paper-based tools (*n* = 121/292), and 11 studies used computer-based tools [58, 99, 106, 118, 143, 184–189], of which only three were online. Seventeen studies used digital devices such as accelerometers for data collection. The remaining studies did not explicitly specify how data were collected.

## Discussion

This systematised review explored the availability and use of tools for assessing adolescent dietary and PA behaviour, knowledge, and awareness in LMICs. We found that self-administered subjective instruments were extensively used to assess adolescent dietary and PA constructs, with a significant focus on the behavioural construct and a minority assessing the knowledge and awareness that underpin such behaviours. Of the 292 studies identified in this study, only 39% used tools assessed for validity or reliability for their context. Tool administration was predominantly self-administered, and 80% of studies used school-based sampling as a population base.

The majority of studies assessed behavioural aspects of adolescent diet and PA constructs (90%), with only 10% assessing knowledge and awareness constructs. Although individual and group behaviour are important entry points for NCD prevention and intervention, socio-cultural environments, knowledge and awareness are key drivers of behaviour change [190]. This highlights a need for the inclusion of knowledge, attitudes and awareness constructs in future epidemiological studies assessing adolescent diet and PA.

Subjective tools were more commonly utilised. This is despite evidence suggesting that the objective tools provide more accurate measures of diet and PA [191–193]. The lower usage of objective tools in LMIC is unsurprising given the relatively high cost of procuring equipment and the need for experienced technicians for data analysis and interpretation. Indeed, global research initiatives, as well as global public health agencies [194] rely on the use of subjective questionnaires to collate information on NCD risk factors trends worldwide owing, in part, to the limited accessibility of objective tools in many parts of the world. Given that subjective tools are currently a more feasible alternative for data collection in LMICs, it is imperative to improve the validation and reliability of existing subjective methods for use in LMICs.

Innovations in the field of mHealth provide a potential avenue to improve objective assessment for both diet and PA. As of 2019, mobile cellular subscriptions was estimated at 105% for people living in LMICs [195]. This extensive mobile coverage in LMIC can be leveraged to collect objective adolescent diet and PA using various techniques [196]. One example is the use of mobile food record apps with fluidical markers (a measured object placed next to the food as a portion size reference) to objectively record types of foods consumed as well as estimate portions sizes [197].

Encouragingly, we noted the emergence of regional and country-specific tools developed and validated for use in LMIC settings. However, only 39% of studies conducted in LMICs used tools validated for use in their specific context. As some of the tools such as the IPAQ (for PA) and YAQ (for diet) have been validated globally, validity may have been assumed for the geographical context. Although adaptation of relevant tools is encouraged, validation of tools is important to reduce errors in outcome reporting that may arise from contextual effects [198]. Regional tools – ATLS [199] and SAYCARE – were developed and validated across countries in the Middle-East and South America to provide a reliable means of comparing food and PA across similar populations. Similarly, TEQ and the MyUM AFFQ were developed for use in Thailand and Malaysia, respectively. Development and validation of diet and PA assessment tools in the contexts in which they are to be used is beneficial as it improves the prospects of accurately capturing local nuances that might be otherwise missed. That said, increased used of locally adapted contextualised tools limits the comparability of research findings across different contexts. This challenge was highlighted in a recent systematic review stating that variations in assessment tools and data collection methods limited the comparability of adolescent girls dietary intake and practices across LMIC regions [200]. Possible strategies for mitigating comparability issues include the inclusion, across all tools, of standardised indicators that conceptually measure the same behavioural constructs [200, 201].

The majority of adolescent participants (75%) in reviewed studies were recruited in school-based studies, with these settings providing access to a captive group of adolescents that can be easily sampled [202, 203]. School-based participant recruitment is suitable in settings, such as European and North American countries, that have low adolescent out of school rates [204]. However, in many LMICs, high school education is not always free, and adolescents from poorer households may not attend school consistently [204]. United Nations Educational, Scientific and Cultural Organization’s reports show that one in three adolescents of school-going age in Sub-Saharan Africa are not in school for various reasons [204]. As such, issues of sampling bias may arise as a school-based sampling may systematically exclude potentially more vulnerable adolescents. Studies in LMICs will therefore need to take this into consideration when developing sampling strategies to ensure greater representation from households, religious groups, and the wider community.

Studies predominantly used self-administered tools for data collection in adolescents. This is based on the assumption that adolescents (aged 10–19 years) have received some formal education and are able to follow instructions and adequately respond to questions during data collection [205]. However, self-administered methods assume a level of literacy that may not always be present in adolescents particularly those living in resource poor regions where access to primary education is not always guaranteed. As such, tools that rely on the adolescent’s ability to read and write, such as dietary or food records, may not be appropriate in populations with low literacy rates [206–208]. In such instances, researcher-led administration of tools to assist participants in completing the data collection task may be more appropriate.

Lastly, the majority of studies identified used a cross-sectional study design with only 4% being longitudinal cohort studies. These findings highlighted a paucity of evidence on the validity and reliability of these tools to capture changes in diet and PA constructs over time. The lack of longitudinal evidence is an important knowledge gap particularly in LMIC contexts where rapid urbanisation means food and built environments are dynamic as this may result in more dynamic changes in diet and activity behaviour, knowledge and awareness over time.

### Study limitations

Qualitative studies on adolescent dietary and PA were not included in this review because of the limited generalisability of qualitative research findings [209]. Consequently, it is possible that relevant research in the area of knowledge and awareness (traditionally measured using qualitative methods) was missed. Therefore, future research may seek to consolidate quantitative and qualitative methods used to assess adolescent diet and PA. Full text articles were only included for review if they were written in English or translated versions were readily available. As a result, we may have excluded contextually relevant studies published in other languages. As the aim of this review was to synthesise existing evidence on validated instruments used to measure diet and physical activity in the context of adolescents in low and middle-income settings, a synthesis of findings on patterns of diet and physical activity behaviour from papers identified was beyond the scope of this paper and not included.

## Conclusions

Our findings point to a plethora of subjective quantitative epidemiological tools for assessing adolescent diet and PA in LMICs, predominantly measuring the behaviour construct. Research studies in LMICs provide useful insights on diet and PA trends, however, more research is required on the reliability and validity of these tools for use, in both cross sectional and longitudinal studies, in LMIC contexts. Furthermore, we recommend that future LMIC study protocols consider adolescent population demographics and socio-economic contexts and adjust sampling and tool administration strategies accordingly. Lastly, this review highlights a need for the inclusion of measures of adolescent knowledge and awareness on diet and PA in future research in LMICs for a more comprehensive understanding of the epidemiology of diet and PA in adolescents and to inform contextually relevant interventions for NCD prevention at this critical life stage.

## Supplementary Information

**Additional file 1.**

**Additional file 2.**

**Additional file 3.**

## Data Availability

All data generated or analysed during this study are included in this published article [and its supplementary information files].

## Abbreviations

LMIC: Low to middle-income countries
NCD: Non-communicable disease
PA: Physical activity
HIC: High-income country
IPAQ: International Physical Activity Questionnaire
PAQ: Physical Activity Questionnaire
HBSC: Health Behavior School-aged Children Survey Questionnaire
3DPAR: Three Day Physical Activity Recall
ATLS: Arab Teens Lifestyle Study
SAYCARE: South American Youth/Child Cardiovascular and Environment
GSHS: Global School Health Survey
NTFFQ: Nutrition Transition Food Frequency Questionnaire
AEHQ: The Arabic Eating Habits Questionnaire
FFQ: Food frequency questionnaire
FGP: Food Guide Pyramid
TPACS-SQ: Thailand PA Children Survey
VAPARQ: Vietnamese Adolescent PA Recall Questionnaire
PeNSE: Brazil National Adolescent School-based Health Survey
CAPANS: Child and Adolescent PA and Nutrition Survey
APARQ: Australia Adolescent Physical Activity Recall Questionnaire
