# Supplementary material for: Assessing adolescent diet and physical activity behaviour, knowledge and awareness in low- and middle-income countries: a systematised review of quantitative epidemiological tools
Source: BMC Public Health. 2022 May 14;22:975. doi: 10.1186/s12889-022-13160-6 (PMC9107740; doi:10.1186/s12889-022-13160-6)
Supplement: Supplementary file 1 — Additional file 1. [file 12889_2022_13160_MOESM1_ESM.docx]

**ADDITIONAL FILE 1**

**Table 1A:** Provisional literature search text and strategy.

| Search number | Search texts |
| --- | --- |
| #1 | epidemiology OR epidemiological |
| #2 | knowledge OR understanding |
| #3 | awareness OR aware |
| #4 | #1 OR #2 OR #3 |
| #5 | tool OR tools |
| #6 | assess OR assessment |
| #7 | measure OR measuring OR measurement |
| #8 | instrument OR instruments |
| #9 | #5 OR #6 OR #7 OR #8 |
| #10 | #4 AND #9 |
| #11 | adolescent OR adolescents OR adolescence |
| #12 | teen OR teenagers |
| #13 | young people |
| #14 | youth |
| #15 | #11 OR #12 OR #13 OR #14 |
| #16 | food OR nutrition OR diet |
| #17 | food behaviour OR food behaviour OR eating behaviour OR eating behaviour OR dietary behaviour OR dietary behaviour |
| #18 | food habit OR eating habit OR dietary habit |
| #19 | food practices OR dietary practices |
| #20 | #16 OR #17 OR #18 OR #19 |
| #20 | #10 AND #15 AND #20 |
| #21 | exercise |
| #22 | physical activity OR activity |
| #22 | walkability OR walk OR walking |
| #24 | #21 OR #22 OR #23 |
| #25 | #10 AND #15 AND #24 |
| #26 | #20 OR #25 |

**Table 1B:** Search strategy undertaken from August to December 2019 (updated in January 2022).

| **Database** | **Results (N)** | **Search** |
| --- | --- | --- |
| Scopus (Medline and Embase) | 3794 | ( ( ( ( ABS ( tools )  OR  ABS ( assessment )  OR  ABS ( measure* )  OR  ABS ( instrument* )  OR  ABS ( questionnaire ) ) )  AND  ( ( ALL ( epidemiolog* )  OR  ALL ( knowledge )  OR  ALL ( aware* )  OR  ALL ( understand* )  OR  ALL ( comprehension* )  OR  ALL ( "public health" ) ) ) )  AND  ( TITLE ( adolesce*  OR  teen*  OR  "young adult"  OR  "youth" ) ) )  AND  ( ( ( TITLE ( food* )  OR  TITLE ( "food behaviour" )  OR  TITLE ( "food behaviour" )  OR  TITLE ( "food practice" )  OR  TITLE ( "food habit" ) ) )  OR  ( ( TITLE ( diet* )  OR  TITLE ( "diet* behaviour" )  OR  TITLE ( "diet* behaviour" )  OR  TITLE ( "diet* practice" )  OR  TITLE ( "diet* habit" ) ) )  OR  ( ( TITLE ( eat* )  OR  TITLE ( "eat* behaviour" )  OR  TITLE ( "eat* behaviour" )  OR  TITLE ( "eat* practice" )  OR  TITLE ( "eat* habit" ) ) ) )  AND  ( EXCLUDE ( EXACTKEYWORD ,  "Eating Disorder" )  OR  EXCLUDE ( EXACTKEYWORD ,  "Energy Intake" )  OR  EXCLUDE ( EXACTKEYWORD ,  "Bulimia" )  OR  EXCLUDE ( EXACTKEYWORD ,  "Human Experiment" )  OR  EXCLUDE ( EXACTKEYWORD ,  "Anthropometry" )  OR  EXCLUDE ( EXACTKEYWORD ,  "Depression" )  OR  EXCLUDE ( EXACTKEYWORD ,  "Anorexia Nervosa" )  OR  EXCLUDE ( EXACTKEYWORD ,  "Binge Eating Disorder" ) )  OR  ( ( ( ( ABS ( tools )  OR  ABS ( assessment )  OR  ABS ( measure* )  OR  ABS ( instrument* )  OR  ABS ( questionnaire ) ) )  AND  ( ( ALL ( epidemiolog* )  OR  ALL ( knowledge )  OR  ALL ( aware* )  OR  ALL ( understand* )  OR  ALL ( comprehension* )  OR  ALL ( "public health" ) ) ) )  AND  ( TITLE ( adolesce*  OR  teen*  OR  "young adult"  OR  "youth" ) ) )  AND  ( ( TITLE ( "PA" )  OR  TITLE ( activity )  OR  TITLE ( exercise )  OR  TITLE ( walk* ) ) )  AND  ( EXCLUDE ( EXACTKEYWORD ,  "Motor Activity" )  OR  EXCLUDE ( EXACTKEYWORD ,  "Pathophysiology" )  OR  EXCLUDE ( EXACTKEYWORD ,  "Anthropometry" )  OR  EXCLUDE ( EXACTKEYWORD ,  "Exercise Test" )  OR  EXCLUDE ( EXACTKEYWORD ,  "Juvenile" )  OR  EXCLUDE ( EXACTKEYWORD ,  "Blood" )  OR  EXCLUDE ( EXACTKEYWORD ,  "Metabolism" )  OR  EXCLUDE ( EXACTKEYWORD ,  "Exercise Therapy" )  OR  EXCLUDE ( EXACTKEYWORD ,  "Kinesiotherapy" )  OR  EXCLUDE ( EXACTKEYWORD ,  "Triacylglycerol" )  OR  EXCLUDE ( EXACTKEYWORD ,  "Adiposity" )  OR  EXCLUDE ( EXACTKEYWORD ,  "Insulin" ) )  AND  ( EXCLUDE ( SUBJAREA ,  "CENG" )  OR  EXCLUDE ( SUBJAREA ,  "VETE" )  OR  EXCLUDE ( SUBJAREA ,  "MATH" )  OR  EXCLUDE ( SUBJAREA ,  "PHYS" )  OR  EXCLUDE ( SUBJAREA ,  "ENER" )  OR  EXCLUDE ( SUBJAREA ,  "MATE" )  OR  EXCLUDE ( SUBJAREA ,  "Undefined" ) ) |
| Web of Science | 1817 | ( ( ( ( ABS ( tools )  OR  ABS ( assessment )  OR  ABS ( measure* )  OR  ABS ( instrument* )  OR  ABS ( questionnaire ) ) )  AND  ( ( ALL ( epidemiolog* )  OR  ALL ( knowledge )  OR  ALL ( aware* )  OR  ALL ( understand* )  OR  ALL ( comprehension* )  OR  ALL ( "public health" ) ) ) )  AND  ( TITLE ( adolesce*  OR  teen*  OR  "young adult"  OR  "youth" ) ) )  AND  ( ( TITLE ( "PA" )  OR  TITLE ( activity )  OR  TITLE ( exercise )  OR  TITLE ( walk* ) ) )  AND  ( EXCLUDE ( EXACTKEYWORD ,  "Motor Activity" )  OR  EXCLUDE ( EXACTKEYWORD ,  "Pathophysiology" )  OR  EXCLUDE ( EXACTKEYWORD ,  "Anthropometry" )  OR  EXCLUDE ( EXACTKEYWORD ,  "Exercise Test" )  OR  EXCLUDE ( EXACTKEYWORD ,  "Juvenile" )  OR  EXCLUDE ( EXACTKEYWORD ,  "Blood" )  OR  EXCLUDE ( EXACTKEYWORD ,  "Metabolism" )  OR  EXCLUDE ( EXACTKEYWORD ,  "Exercise Therapy" )  OR  EXCLUDE ( EXACTKEYWORD ,  "Kinesiotherapy" )  OR  EXCLUDE ( EXACTKEYWORD ,  "Triacylglycerol" )  OR  EXCLUDE ( EXACTKEYWORD ,  "Adiposity" )  OR  EXCLUDE ( EXACTKEYWORD ,  "Insulin" ) )  AND  ( EXCLUDE ( SUBJAREA ,  "CENG" )  OR  EXCLUDE ( SUBJAREA ,  "VETE" )  OR  EXCLUDE ( SUBJAREA ,  "MATH" )  OR  EXCLUDE ( SUBJAREA ,  "PHYS" )  OR  EXCLUDE ( SUBJAREA ,  "ENER" )  OR  EXCLUDE ( SUBJAREA ,  "MATE" )  OR  EXCLUDE ( SUBJAREA ,  "Undefined" ) )  OR  (TOPIC: (epidemiolog*) OR TOPIC: (knowledge) OR TOPIC: (aware*) OR TOPIC: (understand*) OR TOPIC: (public health)) AND (TOPIC: (tool*) OR TOPIC: (assess*) OR TOPIC: (instrument) OR TOPIC: (measur*) OR TOPIC: (evaluat*)) AND (TITLE: (adolescen*) OR TITLE: (teen*) OR TITLE: ("young people") OR TITLE: ("young adult") OR TITLE: (youth)) TITLE: ("PA") OR TITLE: (exercise) OR TITLE: (walk*) |
| EbscoHost (Academic Search Premier, Africa-Wide Information, CINAHL, ERIC, PsycARTICLES, PsycINFO, PsycTESTS) | 4051 | (TI diet* OR TI diet* behaviour OR TI diet* behavior OR TI diet* habit OR TI diet* practice OR TI eat* OR TI eat* behaviour OR TI eat* behavior OR TI eat* habit OR TI eat* practice TI food OR TI food behaviour OR TI food behavior OR TI food habit OR TI food practice OR TI nutrition) AND (epidemiolog* OR knowledge OR aware* OR understand* OR comprehension* OR public health) AND (TI adolescen* OR TI teen* OR TI young people OR TI youth) AND (AB tool OR AB instrument OR AB measure* OR AB assess* OR AB evaluat*)  OR  (TI physical activity OR TI exercise OR TI walk*) AND (epidemiolog* OR knowledge OR aware* OR understand* OR comprehension* OR public health) AND (TI adolescen* OR TI teen* OR TI young people OR TI youth) AND (AB tool OR AB instrument OR AB measure* OR AB assess* OR AB evaluat*) |
