# Supplementary material for: Assessing adolescent diet and physical activity behaviour, knowledge and awareness in low- and middle-income countries: a systematised review of quantitative epidemiological tools
Source: BMC Public Health. 2022 May 14;22:975. doi: 10.1186/s12889-022-13160-6 (PMC9107740; doi:10.1186/s12889-022-13160-6)
Supplement: Supplementary file 2 — Additional file 2. [file 12889_2022_13160_MOESM2_ESM.docx]

**ADDITIONAL FILE 2**

**World Bank Group’s country income classification**

LOW- AND MIDDLE-INCOME COUNTRIES

Afghanistan

Albania

Algeria

American Samoa

Angola

Argentina

Armenia

Azerbaijan

Bangladesh

Belarus

Belize

Benin

Bhutan

Bolivia

Bosnia and Herzegovina

Botswana

Brazil

Bulgaria

Burkina Faso

Burundi

Cabo Verde

Cambodia

Cameroon

Central African Republic

Chad

China

Colombia

Comoros

Congo, Dem. Rep

Congo, Rep.

Costa Rica

Côte d'Ivoire

Cuba

Djibouti

Dominica

Dominican Republic

Ecuador

Egypt, Arab Rep.

El Salvador

Equatorial Guinea

Eritrea

Eswatini

Ethiopia

Fiji

Gabon

Gambia, The

Georgia

Ghana

Grenada

Guatemala

Guinea

Guinea-Bissau

Guyana

Haiti

Honduras

India

Indonesia

Iran, Islamic Rep.

Iraq

Jamaica

Jordan

Kazakhstan

Kenya

Kiribati

Korea, Dem. People's Rep.

Kosovo

Kyrgyz Republic

Lao PDR

Lebanon

Lesotho

Liberia

Libya

Madagascar

Malawi

Malaysia

Maldives

Mali

Marshall Islands

Mauritania

Mexico

Micronesia, Fed. Sts.

Moldova

Mongolia

Montenegro

Morocco

Mozambique

Myanmar

Namibia

Nepal

Nicaragua

Niger

Nigeria

North Macedonia

Pakistan

Papua New Guinea

Paraguay

Peru

Philippines

Russian Federation

Rwanda

Samoa

São Tomé and Principe

Senegal

Serbia

Sierra Leone

Solomon Islands

Somalia

South Africa

South Sudan

Sri Lanka

St. Lucia

St. Vincent and the Grenadines

Sudan

Suriname

Syrian Arab Republic

Tajikistan

Tanzania

Thailand

Timor-Leste

Togo

Tonga

Tunisia

Turkey

Turkmenistan

Tuvalu

Uganda

Ukraine

Uzbekistan

Vanuatu

Venezuela, RB

Vietnam

West Bank and Gaza

Yemen, Rep.

Zambia

Zimbabwe

HIGH INCOME COUNTRIES

Andorra

Antigua and Barbuda

Aruba

Australia

Austria

Bahamas, The

Bahrain

Barbados

Belgium

Bermuda

British Virgin Islands

Brunei Darussalam

Canada

Cayman Islands

Channel Islands

Chile

Croatia

Curaçao

Cyprus

Czech Republic

Denmark

Estonia

Faroe Islands

Finland

France

French Polynesia

Germany

Gibraltar

Greece

Greenland

Guam

Hong Kong SAR, China

Hungary

Iceland

Ireland

Isle of Man

Israel

Italy

Japan

Korea, Rep.

Kuwait

Latvia

Liechtenstein

Lithuania

Luxembourg

Macao SAR, China

Malta

Mauritius

Monaco

Nauru

Netherlands

New Caledonia

New Zealand

Northern Mariana Islands

Norway

Oman

Palau

Panama

Poland

Portugal

Puerto Rico

Qatar

Romania

San Marino

Saudi Arabia

Seychelles

Singapore

Sint Maarten (Dutch part)

Slovak Republic

Slovenia

Spain

St. Kitts and Nevis

St. Martin (French part)

Sweden

Switzerland

Taiwan, China

Trinidad and Tobago

Turks and Caicos Islands

United Arab Emirates

United Kingdom

United States

Uruguay

Virgin Islands (U.S.)
