# Supplementary material for: Assessing adolescent diet and physical activity behaviour, knowledge and awareness in low- and middle-income countries: a systematised review of quantitative epidemiological tools
Source: BMC Public Health. 2022 May 14;22:975. doi: 10.1186/s12889-022-13160-6 (PMC9107740; doi:10.1186/s12889-022-13160-6)
Supplement: Supplementary file 3 — Additional file 3. [file 12889_2022_13160_MOESM3_ESM.docx]

**ADDITIONAL FILE 3**

**Characterisation of studies assessing diet or physical activity in low- and middle-income countries (LMIC)**

**Table 3A:** Studies that assessed both physical activity and diet in low- and middle-income countries (LMICs). Studies are characterised by geographical scope (international, regional, or country-specific). All studies used subjective tools.

| **Authors** | **Country** | **Study Design** | **Sample** | **Tool type** | **Tool name** | **Initial population** | **Administration** | | **Construct** | **Validation/Reliability** |  |
| --- | --- | --- | --- | --- | --- | --- | --- | --- | --- | --- | --- |
| **International** | | | | | | | | | | |  |
| Christofaro et al. 2016 (1) | Brazil | Cross-sectional | 1267 students (14 to 17 years) | Questionnaire | Baecke questionnaire / Not specified | General population | Self | Usual dietary practices / Habitual physical activity | | Yes - Test-retest reliability |  |
| Farah Wahida et al. 2011 (2) | Malaysia | Cross-sectional | 360 students (13 to 14 years) | Questionnaire | Physical Activity Questionnaire for Older Children - PAQ-C/ Eating Attitudes Test - EAT | Adolescent | Self | Usual dietary practices / Eating attitudes | | No |  |
| Hatami et al. 2014 (3) | Iran | Cross-sectional | 1157 students (10 to 18 years) | Questionnaire | Food Frequency Questionnaire - FFQ / Physical Activity Questionnaires for Adolescents - PAQ-A | Adolescent | Self | Usual food consumption / Habitual physical activity | | Yes - Content validity |  |
| Kafeshani et al. 2015 (4) | Iran | Cross-sectional | 14880 students (6 to 18 years) | Questionnaire | Global School-based Student Health Survey - WHO-GSHS | Adolescent | Interviewer | Usual dietary practices / Habitual physical activity and sedentary behaviour | | Yes - Content validity/ Test-retest reliability |  |
| Masoomi et al. 2019 (5) | Iran | Cross-sectional | 348 students (13.9 +/- 2.7 years) | Questionnaire | Food Frequency Questionnaire - FFQ / International Physical Activity Questionnaire - IPAQ | General Population | Self | Usual food consumption / habitual physical activity | | Yes - Face validity/ Content validity/ Construct validity |  |
| Safiri et al. 2016 (6) | Iran | Cross-sectional | 14880 students (12.47 +/-3.36 years) | Questionnaire | Global School-based Student Health Survey - WHO-GSHS | Adolescent | Interviewer | Usual food consumption (including fast food) / Habitual physical activity | | Yes - Content validity/ Test-retest reliability |  |
| Saikia et al. 2016 (7) | India | Cross-sectional | 800 students (10 to 14 years) | Questionnaire | Food Frequency Questionnaire - FFQ/ Global Physical Activity Questionnaire - GPAQ | General adult population | Interviewer | Usual food consumption / Habitual physical activity | | No |  |
| Prioreschi et al. 2017 (8) | South Africa | Cross-sectional | 509 adolescents (18 to 23 years) | Questionnaire | GPAQ - Global Physical Activity Questionnaire - GPAQ/ Eating Attitudes Test - EAT | General population | Self | Habitual physical activity / Sedentary behaviour | | No |  |
| McArthur et al. 2008 (9) | Latin America | Cross-sectional | 1279 students (8th and 9th grade) | Questionnaire | Food Frequency Questionnaire - FFQ/ Activity Frequency Questionnaire - AFQ | General Population | Self | Usual food consumption/ habitual physical activity | | No |  |
| **Regional** | | | | | | | | | | |  |
| Smith et al. 2007 (10) | Tonga | Cross-sectional | 443 students (11 to 16 years) | Questionnaire | Health Behaviour and Lifestyle of Pacific Youth questionnaire - HBLPY | Adolescent | Self | Usual dietary practices / Habitual physical activity | | No |  |
| Tayyem 2014 (11) | Jordan | Cross-sectional | 735 students (14 to 18 years) | Questionnaire | Arab Teens Lifestyle Study (ATLS) | General population | Self | Dietary habits and physical activity | | No |  |
| Musaiger et al. 2014 (12) | Iraq | Cross-sectional | 723 students (15 to 18 years) | Questionnaire | Arab Teenage Lifestyle questionnaire - ATLS | Adolescent | Self | Sedentary and dietary behaviour / Usual food consumption | | Yes - Criterion validity (pedometer)/ Test-retest reliability |  |
| **Country-specific** | | | | | | | | | | | |
| Citozi et al. 2013 (13) | | Albania | Cross-sectional | 340 students (15.2 ± 0.5 years) | Questionnaire | Not specified | Not specified | Self | Eating habits/ Physical activity/ Healthy and unhealthy dietary habits and food/ Self-efficacy/ Barriers to change/ Nutritional knowledge. | | No |
| Abdullah et al. 2016 (14) | | Malaysia | Cross-sectional | 454 students (12 to 19 years) | Questionnaire | computer-based-PA questionnaire - cPAQ/ Food Frequency Questionnaire -FFQ | Not specified | Self | Usual dietary practices / Habitual physical activity | | No |
| Chansukree et al. 2017 (15) | | Thailand | Cross-sectional | 1200 students | Questionnaire | Not specified | Not specified | Self | Eating habits/ Habitual physical activity | | No |
| Flor-Garrido et al. 2016 (16) | | Ecuador | Cross-sectional | 314 students (12 to 19 years) | Questionnaire | Not specified | Not specified | Self | Fruit and vegetable consumption/ Habitual physical activity | | No |
| Kelishadi et al. 2016 (17) | | Iran | Cross-sectional | 1992 students (11 to 18 years) | Questionnaire | Not specified | Not specified | Interviewer | Usual dietary practices / Habitual physical activity | | No |
| Musaiger et al. 2013 (18) | | Arab countries* | Cross-sectional | 4698 students (15 to 18 years) | Questionnaire | Not specified | Not specified | Self | Habitual physical activity / Usual food consumption | | Yes – Inter-rater reliability |
| Trang et al. 2012 (19) | | Vietnam | Cohort | 759 students (11.8 +/-0.6 years) | Questionnaire | Food Frequency Questionnaire - FFQ/ Vietnamese-Adolescent Physical Activity Recall Questionnaire - V-APARQ/ Accelerometer | Adolescent | Interviewer | Usual food consumption / Habitual physical activity | | No |
| Musaiger et al. 2016 (20) | | Sudan | Cross-sectional | 945 students (14 to 18 years) | Questionnaire | Not specified | Not specified | Self | Usual food consumption / Habitual physical activity | | Yes - Content validity/ Test-retest reliability |
| De Oliveira et al. 2018 (21) | | Brazil | Cross-sectional | 14653 students (14.0 +/- 1.0 years) | Questionnaire | National School Health Survey 2009 (Online) | Adolescent | Self | dietary behaviour / screen time / physical activity | | No |
| Costa et al 2021 (22) | | Brazil | Cross-sectional | 615 students (14 to 18 years) | Questionnaire/Accelerometery | Estudo Longitudinal do Estilo de Vida de Adolescentes (ELEVA) / ActiGraph | Adolescent | Self | Sedentary behaviour / Leisure time physical activity / Dietary patterns | | Yes - Factor analysis |
| Zalilah et al 2006 (23) | | Malaysia | Cross-sectional | 6555 adolescents (11 to 15 years) | Food and physical activity records | 3 Day food record/ 3 Day physical activity record | Not specified | Self | Usual dietary intake and physical activity | | Not specified |
| Djordjevic-Nikic et al. 2013 (24) | | Serbia | Cross-sectional | 330 students (16 years) | Questionnaire | Not specified | Not specified | Interviewer | PA / Eating habits/ Self efficacy/ barriers to change / Nutritional knowledge | | Yes - Internal consistency |

***** Algeria, Jordan, Kuwait, Libya, Palestine, Syria, and the United Arab Emirates (UAE)

**Table 3B:** Studies that only assessed physical activity constructs in low- and middle-income countries (LMICs). Studies are characterised by type (subjective or objective) and geographical scope (international, regional, or country-specific).

| **Author(s)** | **Country** | **Study** | **Sample** | **Tool** | **Instrument name** | **Initial Population** | **Administration** | **Construct** | **Validation** | |
| --- | --- | --- | --- | --- | --- | --- | --- | --- | --- | --- |
| **Subjective tools** |  |  |  |  |  |  |  |  |  | |
| **International** | | | | | | | | | | |
| Asare et al. 2015 (25) | Ghana | Cross-sectional | 296 students (13 to 18 years) | Questionnaire | Physical Activity Questionnaire for Adolescents - PAQ-A | Adolescents | Self | Habitual physical activity | | No |
| Azizi-Soleiman et al. 2016 (26) | Iran | Cross-sectional | 14880 students (6 to 18 years) | Questionnaire | Global School-based Health Survey - GSHS | Adolescents | Self | Habitual physical activity | Yes - Face validity / Content validity /Test-retest reliability | |
| Chen et al. 2018 (27) | China | Cross-sectional | 50090 students (10 to 18 years) | Questionnaire | Health Behaviour School-aged Children survey questionnaire - HBSC | Adolescents | Self | Moderate and vigorous physical activity | No | |
| D'Alonzo et al. 2007 (28) | Costa Rica | Cross-sectional | 19 adolescents (12 to 19 years) | Questionnaire | Past Year Leisure-Time Physical Activity Questionnaire | Adolescents | Self | Habitual physical activity | No | |
| Dan et al. 2007 (29) | Malaysia | Cross-sectional | 400 adolescents (13 years) | Questionnaire | Physical Activity Questionnaire for Older Children - PAQ-C | Adolescents | Self | Habitual physical activity | No | |
| Dave et al. 2017 (30) | India | Cross-sectional | 3337 students (10 to 19 years) | Questionnaire | Physical Activity Questionnaire for Adolescents - PAQ-A | Adolescents | Self | Habitual physical activity | No | |
| Guimaraes et al. 2013 (31) | Brazil | Cross-sectional | 122 students (12 to 17 years) | Questionnaire | Adolescent Sedentary Activity Questionnaire - ASAQ | Adolescents | Self | Habitual physical activity and sedentary behaviour | Yes - Internal Consistency | |
| Fazah et al. 2010 (32) | Lebanon | Cross-sectional | 1000 students (14 to 18 years) | Questionnaire | Paediatric Quality of Life Inventory - PedsQL | Adolescents | Self | Habitual physical activity | No | |
| Glozah et al. 2015 (33) | Ghana | Cross-sectional | 770 students (14 to 21 years) | Questionnaire | Revised Personal Lifestyle Questionnaire - RPLQ | Adolescents | Self | Type of physical activity | No | |
| Greca et al. 2016 (34) | Brazil | Cross-sectional | 306 students (12.8 +/-1.8 years) | Questionnaire | Physical Activity Questionnaire for Children - PAQ-C | Adolescents | Self | Habitual physical activity | No | |
| Jabeen et al. 2018 (35) | Pakistan | Cross-sectional | 216 students (10 to 17 years) | Questionnaire | Physical Activity Questionnaire for older children Questionnaire - PAQ-C | Adolescents | Self | Habitual physical activity | No | |
| Kundapur et al. 2017 (36) | India | Cross-sectional | 300 students (12 to 16 years) | Questionnaire | PA questionnaire for adolescents - PAQ-A | Adolescents | Self | Habitual physical activity | No | |
| Lachat et al. 2008 (37) | Vietnam | Cross-sectional | 137 students (16 years) | Questionnaire | International Physical Activity Questionnaire - IPAQ / Physical Activity questionnaire for adolescents - PAQ-A | Adolescents | Self | Habitual physical activity | Yes - Test-retest reliability/ Criterion validity/ Criterion validity (Accelerometer) | |
| .Lennox et al. 2008 (38) | South Africa | Cross-sectional | 252 students (15 years) | Questionnaire | Previous Day Physical Activity Recall - PDPAR | Adolescents | Self | Habitual physical activity | No | |
| Li et al. 2007 (39) | China | Cross-sectional | 1804 students (11 to 17 years) | Questionnaire | Adolescents Physical Activity Recall Questionnaire - APARQ | Adolescents | Self | Habitual physical activity and sedentary behaviour | No | |
| Mamabolo et al. 2007 (40) | South Africa | Cross-sectional | 336 students (12 to 18 years) | Questionnaire | Previous Day Physical Activity Recall questionnaires - PDPAR | Adolescents | Interviewer | Habitual physical activity | No | |
| Motamed-Gorji et al. 2019 (41) | Iran | Cross-sectional | 25000 students (6 to 18 years) | Questionnaire | Physical Activity Questionnaire for Adolescents - PAQ-A | Adolescents | Self | Habitual physical activity and sedentary behaviour | Yes - Face validity/ Content validity | |
| Oyeyemi et al. 2014 (42) | Nigeria | Cross-sectional | 1006 students (12 to 19 years) | Questionnaire | Activity Questionnaire for Adolescents and Young Adults - AQuAA | Adolescents | Self | Habitual physical activity | No | |
| Oyeyemi et al. 2016 (43) | Nigeria | Cross-sectional | 1006 students (12 to 18 years) | Questionnaire | Activity Questionnaire for Adolescents and Young Adults - AQuAA | Adolescents | Self | Habitual physical activity | Yes - Test-retest reliability | |
| Ramezankhani et al. 2016 (44) | Iran | Cross-sectional | 308 students (13.86 ± 1.3 years) | Questionnaire | Standard Physical Activity Questionnaire - SPAQ | Adolescents | Self | Habitual physical activity / Perceived benefits and barriers for regular physical activity | No | |
| Sanaeinasab et al. 2013 (45) | Iran | Cross-sectional | 1551 students (12 to 14 to years) | Physical Activity Record | Child/Adolescent Activity Log - CAAL | Adolescents | Self | Previous day physical activity | No | |
| Shokrvash et al. 2013 (46) | Iran | Cross-sectional | 402 students (12.93 +/-0.49 years) | Questionnaire | Adolescent Physical Activity and Recall Questionnaire - APARQ | Adolescents | Self | Habitual physical activity / Physical activity self-efficacy/Family support specific to physical activity | Yes - test-retest reliability/ internal consistency /face validity | |
| Taymoori et al. 2012 (47) | Iran | Longitudinal | 844 students (16.42 +/- 1.738 years) | Physical Activity Record | Child/Adolescent Activity Log - CAAL | Adolescents | Self | Physical activity type and duration | Yes - Test-retest reliability | |
| Abasi et al. 2016 (48) | Iran | Cross-sectional | 734 students (15 to 19 years) | Questionnaire | International Physical Activity Questionnaire - IPAQ | Adults | Self | Habitual physical activity | Yes - Face validity / Content validity / Reliability (Confirmatory Factor Analysis) | |
| Fortes et al. 2013 (49) | Brazil | Cross-sectional | 462 students (10 to 19 years) | Questionnaire | International Physical Activity Questionnaire - IPAQ | Adults | Self | Habitual physical activity | Yes - Face validity / Content validity / Reliability (Confirmatory Factor Analysis) | |
| Iskandar et al. 2017 (50) | Malaysia | Cross-sectional | 386 adolescents (15 to 25 years) | Questionnaire | International Physical Activity Questionnaire - IPAQ | Adults | Self | Habitual physical activity | No | |
| Kelishadi et al. 2007 (51) | Iran | Cross-sectional | adolescents (6 to 18 years) | Questionnaire | International Physical Activity Questionnaire - IPAQ | Adults | Self | Habitual physical activity | Yes - Face validity / Content validity/Reliability (Confirmatory Factor Analysis) | |
| Masocha et al. 2018 (52) | South Africa | Cohort | 289 students (14 years) | Questionnaire | International Physical Activity Questionnaire - IPAQ | Adults | Self | Habitual physical activity | No | |
| Paudel et al. 2014 (53) | Nepal | Cross-sectional | 405 students (15 to 20 years) | Questionnaire | International Physical Activity Questionnaire - IPAQ | Adults | Self | Habitual physical activity | No | |
| Pelegrini et al. 2014 (54) | Brazil | Cross-sectional | 892 students (14 to 18 years) | Questionnaire | International Physical Activity Questionnaire - IPAQ | Adults | Self | Habitual physical activity | Yes - Face validity/ Content validity/ Reliability (Confirmatory Factor Analysis) | |
| Straatmann et al. 2019 (55) | Brazil | Cross-sectional | 810 students (11 +/- 0.85 years) | Questionnaire | International Physical Activity Questionnaire - IPAQ | Adults | Self | Habitual physical activity | Yes - Face validity/ Content validity/ Reliability (Confirmatory Factor Analysis) | |
| Suyoto et al. 2016 (56) | Indonesia | Cross-sectional | 77 students (13 to 15 years) | Questionnaire | International Physical Activity Questionnaire - IPAQ | Adults | Self | Habitual physical activity | Yes - Criterion validity (Seven 24hr physical activity recall) | |
| Wang et al. 2013 (57) | China | Cross-sectional | 1021 students (12 to 18 years) | Questionnaire | International Physical Activity Questionnaire - IPAQ | Adults | Self | Habitual physical activity | Yes - Criterion validity (Accelerometer) | |
| Werneck et al. 2018 (58) | Brazil | Cross-sectional | 100839 students (11 to 18 years) | Questionnaire | International Physical Activity Questionnaire - IPAQ | Adults | Self | Habitual physical activity | Yes - Face validity/ Content validity/ Reliability (Confirmatory Factor Analysis) | |
| Yildizer et al. 2019 (59) | Turkey | Cross-sectional | 520 students (14 to 18 years) | Questionnaire | International Physical Activity Questionnaire - IPAQ | Adults | Self | Habitual physical activity | Yes - Criterion validity (Accelerometer)/ Test-retest reliability | |
| Zhu et al. 2019 (60) | China | Cross-sectional | 131859 students (17 to 19 years) | Questionnaire | International Physical Activity Questionnaire - IPAQ | Adults | Self | Habitual physical activity | No | |
| Karaca et al. 2009 (61) | Turkey | Cross-sectional | 1027 students (20.85 +/- 2.01 years) | Questionnaire | Physical Activity Assessment Questionnaire - PAAQ | General population | Self | Habitual physical activity | No | |
| Khan et al. 2017 (62) | Bangladesh | Cross-sectional | 88 students (13 to 17 years) | Questionnaire | Three-day physical activity recall - 3DPAR | General population | Self | Habitual physical activity | No | |
|  |  |  |  |  |  |  |  |  |  | |
| da Silva and Malina 2000 (63) | Brazil | Cross-sectional | 325 students (14 to 15 years) | Questionnaire | Physical Activity Questionnaire (PAQ) | Not specified | Self | Habitual physical activity | Yes - Internal consistency / Test-retest | |
| Hajin-Tilaki and Heidari 2012(64) | Iran | Cross-sectional | 1200 (12 to 17 years) | Questionnaire | Baecke Questionnaire | Not specified | Interviewer | Habitual physical activity | Yes - Reliability | |
| Teo et al 2013(65) | Malaysia | Cross-sectional | 425 adolescents (11 to 16 years) | Questionnaire | computer-based Physical Activity Questionnaire (cPAQ) | General population | Self | Physical activity levels | Yes - Criterion validity (Heart rate monitor) / 7-day physical activity logbook | |
| Yelizarova et al 2020 (66) | Ukraine | Cross-sectional | 415 students (11 to 15 years) | Questionnaire | Quantization de L’Active Physique en Altitude Chez le Enfants (QAPACE) | Adolescents | Self | Physical activity type, multiplicity, duration, and intensity. | Yes - Criterion validity / Test-retest reliability / Internal consistency | |
| Ziaei et al 2020(67) | Iran | Cross-sectional | 1517 students (15 to 17 years) | Questionnaire | Global School-based Student Health Survey (GSHS) | Adolescents | Self | Leisure time physical activity / leisure time sedentary behaviour | Not specified | |
| Andarge et al 2021(68) | Ethiopia | Cross-sectional | 120 students (9 to 12th grade) | Questionnaire | Physical Activity Questionnaire for Adolescents (PAQ–A) | Adolescents | Self | Habitual physical activity | Yes - Test-retest reliability / Inter-item correlations | |
| Mohammed et al. 2020 (69) | Ethiopia | Cross-sectional | 580 students (13 to 19 years) | Questionnaire | International physical activity questionnaire (IPAQ) | Adolescents | Self | Physical activity frequency and intensity | Yes - Internal consistency | |
| Peralta et al 2020 (70) | 63 LMIC countries | Cross-sectional | 187934 adolescents (11 to 16 years) | Questionnaire | Global School-based Student Health Survey (GSSH) | Adolescents | Self | Active commuting to school | Yes - Criterion validity (accelerometer) | |
| Nikolic et al 2020(71) | South Africa | Cross-sectional | 401 students (15to17 years) | Questionnaire | International Physical Activity Questionnaire (IPAQ) | Adolescents | Self | Habitual physical activity | Yes - Criterion validity (pedometer) | |
| Peltzer et al. 2010 (72) | African countries* | Cross-sectional | 24593 students (13 to 15 years) | Questionnaire | Global School-Based Health Survey - GSHS | Not specified | Self | Habitual moderate and vigorous physical activity | Yes - Content validity; Test-retest reliability | |
| **Regional** | | | | | | | | | | |
| Abu-Mweis et al. 2014 (73) | Jordan | Cross-sectional | 735 students (14 to 20 years) | Questionnaire | Arab Teens Lifestyle Study Questionnaire -ATLS | Adolescents | Self | Habitual physical activity and sedentary behaviour | Yes - Criterion validity (pedometer) / Test-retest reliability | |
| El Achhab et al. 2018 (74) | Morocco | Cross-sectional | 346 students (14 to 19 years) | Questionnaire | Arab Teens Lifestyle Study Questionnaire - ATLS | Adolescents | Self | Habitual physical activity | Yes - Criterion validity (pedometer) / Test-retest reliability | |
| Hamrani et al. 2015 (75) | Morrocco | Cross-sectional | 669 students (15.0 to19.9 years) | Questionnaire | Arab Teenage Lifestyle questionnaire - ATLS | Adolescents | Self | Physical activity and sedentary behaviour (travel, within-household, fitness, and sports activities) | Yes - Criterion validity (pedometer) / Test-retest reliability | |
| Nascimento-Ferreira et al. 2018 (76) | South America | Cross-sectional | 120 students (3 to 18 years) | Questionnaire | South American Youth/Child Cardiovascular and Environment Physical Activity Questionnaire - SAYCARE | Adolescents | Self | Habitual physical activity | Yes - Criterion validity (Accelerometer)/ Test-retest reliability | |
| Zieff et al. 2006 (77) | Brazil | Cross-sectional | 55 students (14 to 18 years) | Questionnaire | Unnamed | Adolescents | Self | Adolescent understanding about the importance of PA in relation to health | No | |
| **Country-specific** | | | | | | | | | | |
| Amornsriwatanakul et al. 2017 (78) | Thailand | Cross-sectional | 13255 students (6 to 17 years) | Questionnaire | Thailand Physical Activity Children Survey - TPACS-SQ | Adolescents | Self | Habitual physical activity | Yes - Test-retest reliability | |
| Christoph et al. 2017 (79) | Uganda | Cross-sectional | 148 students (11 to 16 years) | Questionnaire | Uganda Global School-Based Student Health Survey GSHS - GSHS | Adolescents | Self | Habitual physical activity and sedentary behaviour | Yes - Content validity; Test-retest reliability | |
| de Lima et al. 2018 (80) | Brazil | Cross-sectional | 1103 students (14 to 19 years) | Questionnaire | Brazilian version of the Youth Risk Behaviour Surveillance questionnaire - YRBSS | Adolescents | Interviewer | Habitual physical activity | No | |
| Florindo et al. 2006 (81) | Brazil | Cross-sectional | 94 students (11 to 16 years) | Questionnaire | Unnamed | Adolescents | Interviewer | Habitual physical activity | Yes - Test-retest reliability | |
| Gharbia et al. 2014 (82) | Tunisia | Cross-sectional | 142 students (10 to 19 years) | Questionnaire | Physical Activity Frequency Questionnaire | Adolescents | Interviewer | Habitual physical activity | Yes - Criterion validity (Heart Rate Monitor and physical activity recall) | |
| Ghavamzadeh et al. 2013 (83) | Iran | Cross-sectional | 2498 students (11 to 20 years) | Questionnaire | Unnamed | Adolescents | Self | Habitual physical activity | No | |
| Hong et al. 2012 (84) | Vietnam | Cross-sectional | 165 students (12.7 +/-0.6 years) | Questionnaire | Vietnamese Adolescent Physical Activity Recall Questionnaire - VAPARQ | Adolescents | Self | Habitual physical activity in summer and winter terms | Yes - Content validity / Test-retest validity | |
| Kee et al. 2011 (85) | Malaysia | Cross-sectional | 785 students (14 to 17 years) | Questionnaire | Malay Version - School Health Action, Planning and Evaluation System - SHAPES | Adolescents | Self | Physical activity frequency and duration | No | |
| Micklesfield et al. 2014 (86) | South Africa | Cross-sectional | 381 students (11 to 15 years) | Questionnaire | Unnamed | Adolescents | Self | Habitual physical activity and sedentary behaviour | No | |
| Nguyen et al. 2012 (87) | Vietnam | Longitudinal | 759 students (11.8 +/- 0.6 years) | Questionnaire | Vietnamese Adolescent Physical Activity Recall Questionnaire Physical Activity Questionnaire - V-APARQ | Adolescents | Self | Habitual physical activity behaviour | Yes - Content validity / Test-retest validity | |
| Prista et al. 1997 (88) | Mozambique | Cross-sectional | 909 adolescents (8 to 15 years) | Questionnaire | Unnamed | Adolescents | Self | Habitual physical activity | Yes - Criterion validity (24hr observation)/ Test-retest reliability | |
| Silva et al. 2019 (89) | Brazil | Cross-sectional | 12220 students (11 to 19 years) | Questionnaire | PeNSE questionnaire | Adolescents | Self | Habitual physical activity | Yes - Criterion validity (24hr physical activity recall) | |
| Su et al. 2014 (90) | Malaysia | Cross-sectional | 1361 students (13 years) | Questionnaire | Malay version of the Physical Activity Questionnaire for Older Children - PAQ-C | Adolescents | Self | Habitual physical activity | No | |
| Teo et al. 2014 (91) | Malaysia | Cross-sectional | 456 students (12 to 19 years) | Questionnaire | Computer-based-PA questionnaire - cPAQ | Adolescents | Self | Habitual physical activity | Yes - Criterion validity (Heart Rate Monitor, Physical Activity Logbook)/ Test-retest reliability | |
| Verstraeten et al. 2013 (92) | Ecuador | Cross-sectional | 302 students (11 to 15 years) | Physical Activity Record | Physical Activity Record | Adolescents | Self | Habitual physical activity | Yes - Criterion validity (Accelerometer) | |
| Afrifa-Anane et al. 2015 (93) | Ghana | Cross-sectional | 201 adolescents (14 to 24 years) | Questionnaire | Edulink Urban Health and Poverty project questionnaire | General population | Self | Leisure time physical activity | No | |
| Barbosa et al. 2016 (94) | Colombia | Cross-sectional | 1840 students (8 to 16 years) | Questionnaire | Quantification de l’Activite Physique en Altitude Chez les Enfants - QAPACE | General population | Self | Habitual physical activity | No | |
| Delshad et al. 2015 (95) | Iran | Cross-sectional | 80 students (12 to 18 years) | Questionnaire | Modifiable Activity Questionnaire - MAQ | General population | Self | Leisure time physical activity | No | |
| TudorLocke et al. 2003 (96) | China | Cross-sectional | 2675 students (11.5 +/-3.3 years) | Questionnaire | China Health and Nutrition Survey - CHNS | General population | Self | Habitual physical activity | No | |
| Bastos 2008 (97) | Brazil | Cross-sectional | 857 adolescents (10 to 19 years) | Questionnaire | Unnamed | Not specified | Interviewer | Habitual physical activity (transport and leisure time physical activity) | Yes - Criterion validity (pedometer) / Test-retest reliability | |
| Bastos et al. 2008 (98) | Brazil | Cross-sectional | 857 adolescents | Questionnaire | Not specified | Adolescents | Interviewer | Habitual physical activity | Yes - Criterion validity (pedometer) | |
| Micklesfield et al 2014(99) | South Africa | Cross-sectional | 381 adolescents (11 to 12, 14 and 15 years) | Questionnaire | Physical Activity Questionnaire (PAQ) | General population | Interviewer | Habitual physical activity | Yes - Test-retest | |
| Mehreen et al. 2020 (100) | India | Cross-sectional (validation study) | 104 adolescents (10 to 17 years) | Questionnaire | Madras Diabetes Research Foundation – Physical Activity Questionnaire for Children and Adolescents (MPAQ©) | Adolescents | Self | General physical activity | Yes - Test-retest reliability / Criterion validity (accelerometer) | |
| Ferrari et al 2020 (101) | Brazil | Cross-sectional | 2682 students (14 to 15 years) | Questionnaire | National Survey of School Health - Physical activity section | Adolescents | Self | Frequency and duration of active transport / Leisure time physical activity / Participation in physical education classes | No | |
| Coll et al. 2014 (102) | Brazil | Cross-sectional | 743 adolescents (10 to 19 years) | Questionnaire | Unnamed | Not specified | Interviewer | Habitual physical activity and sedentary behaviour | Yes - Test-retest reliability | |
| **Objective tools** |  |  |  |  |  |  |  |  |  | |
| **International** | | | | | | | | | | |
| Alberico et al. 2018 (103) | Brazil | Cross-sectional | 381 adolescents (12 to 17 years) | Accelerometer | ActiGraph Accelerometer | General population | Self | Physical activity type and duration | No | |
| Benefice et al. 1999 (104) | Senegal | Cross-sectional | 40 adolescents (13.3 +/-0.5 years) | Accelerometer / Direct observation | CSA Accelerometer / Direct observation | General population | Observer/ Self | Physical activity duration and intensity | No | |
| Benefice et al. 2001 (105) | Senegal | Cohort | 40 adolescents (13.3 +/-0.5 years) | Accelerometer | CSA Accelerometer | General population | Self | Physical activity duration and intensity | No | |
| Cook 2015 (106) | South Africa | Cross-sectional | 178 adolescents (13.7 to 18.0 years) | Accelerometer | Unnamed | General population | Self | Daily step count | No | |
| Corder et al. 2007(107) | India | Cross-sectional | 30 students (15.8 +/-0.6 years) | Accelerometer | ActiGraph Accelerometer | General population | Self | Physical activity type and duration | No | |
| da Costa et al. 2018 (108) | Brazil | Cross-sectional | 567 students (12.3 +/-1.3 years) | Accelerometer | ActiGraph Accelerometer | General population | Self | Physical activity duration and intensity | No | |
| Konharn et al. 2015 (109) | Thailand | Cross-sectional | 186 students (13 to 18 years) | Accelerometer | ActiGraph Accelerometer | General population | Self | Physical activity duration and intensity | No | |
| Nie et al. 2019 (110) | Tibet | Cross-sectional | 397 students (9 to 18 years) | Accelerometer | ActiGraph Accelerometer | General population | Self | Physical activity duration and intensity | No | |
| Ojiambo et al. 2012 (111) | Kenya | Cross-sectional | 247 students (12 to 16 years) | Accelerometer | ActiGraph Accelerometer | General population | Self | Physical activity duration and intensity | No | |
| Sayre et al. 2019 (112) | Kenya | Cross-sectional | 40 adolescents (14 to 18 years) | Accelerometer | ActiGraph Accelerometer | General population | Self | Physical activity duration and intensity | No | |
| Naseer 2020 (113) | Pakistan | Cross-sectional | 265 adolescents (13 to 16 years) | Pedometer | Ymax Digi Walker SW-200 | Not specified | self | Number of steps | Yes - internal consistency | |
| Wushe et al. 2014 (114) | South Africa | Cross-sectional | 226 students (14 to 18 years) | Heart rate monitor | Actiheart® | General population | Self | Habitual physical activity | No | |
| **Combined objective and subjective tools** | | | | | | | | | | |
| **International** | | | | | | | | | | |
| Corder et al. 2010 (115) | India | Cross-sectional | 30 students (15.8 +/-0.59 years) | Accelerometer / Questionnaire / DLW | ActiGraph Accelerometer / Youth Physical Activity Questionnaire - YPAQ | General population / Youth | Self | Habitual physical activity | No | |
| da Silva et al. 2017 (116) | Brazil | Cross-sectional | 3379 adolescents (16 to 20 years) | Accelerometer / Questionnaire | GENEActiv Accelerometer / International Physical Activity Questionnaire - IPAQ | General population/ Adolescents | Self | Habitual physical activity | Yes - Face validity / Content validity / Reliability (Confirmatory Factor Analysis) | |
| Prista et al. 2009 (117) | Mozambique | Cross-sectional | 256 students (6 to 16 years) | Accelerometer / Questionnaire | ActiGraph Accelerometer | General population/ Adolescents | Self | Habitual physical activity | **Yes –** Criterion validity (Indirect colorimetry / Doubly labelled water) | |
| Hallal et al. 2013 (118) | Brazil | Cross-sectional | 25 students (13 years) | Accelerometer / Questionnaire / DLW | ActiGraph/ Unnamed | General population | Interviewer | Physical activity type and duration | Yes - Test-retest reliability / Concurrent validity | |
| **Country-specific** | | | | | | | | | | |
| Prista et al. 2000 (119) | Mozambique | Cross-sectional | 20 students (10-15 years) | PA recall | Unnamed | Youth | Self | Habitual physical activity | Yes – Criterion validity (direct observation) | |
| Tudor-Locke et al. 2003 (120) | Philippines | Cross-sectional | 2083 adolescents (14 to 16 years) | Accelerometer / Questionnaire / 24hr recall | Cebu Longitudinal Health and Nutrition Survey - CLHNS / Caltrac Accelerometer / 24hr physical activity recall | Adults | Self | Habitual physical activity | No | |
| Aniza et al 2009 (121) | Malaysia | Cross-sectional | 519 students (14 to 16 years) | Questionnaire | International Physical Activity Questionnaire (IPAQ) | Not specified | Self | Habitual physical activity | No | |
| Gouthon et al 2007 (122) | Benin | Cross-sectional | 687 (13 to 23 years) | Questionnaire | Not specified | Not specified | Self | Habitual physical activity | Yes - Test-retest | |
| Hovsepian et al 2019 (123) | Iran | Cross-sectional | 14880 (6 to 18 years) | Questionnaire | Childhood and Adolescence Surveillance and Prevention of Adult Non- communicable Disease (CASPIAN-IV) Survey | Adolescent | Self | Habitual physical activity / Sedentary behaviour | Not specified | |
|  |  |  |  |  |  |  |  |  |  | |
| *Botswana, Kenya, Namibia, Senegal, Swaziland, Uganda, Zambia, and Zimbabwe | | | | | | | | | | |

**Table 3C:** Studies that only assessed dietary constructs in low- and middle-income countries. Studies are characterised by geographical scope (international, regional, or country-specific). All studies used subjective tools.

| **Study** | **Country** | **Study Design** | **Sample** | **Instrument** | **Instrument name** | **Initial population** | **Administration** | **Construct** | **Validation** |
| --- | --- | --- | --- | --- | --- | --- | --- | --- | --- |
| **International** | | | | | | | | | |
| Akman et al. 2010 (124) | Turkey | Cross-sectional | 625 students (11 to 15 years) | Questionnaire | Questionnaire of Eating Patterns - QEP | Adolescents | Self | Eating patterns/ Knowledge about healthy eating | No |
| Araki et al. 2011 (125) | Brazil | Cross-sectional | 1280 students (14 to 17 years) | Questionnaire | Adolescent Eating Attitudes Questionnaire - AEAQ | Adolescents | Self | Usual dietary practices | No |
| Can et al. 2008 (126) | Turkey | Cross-sectional | 390 students (17 +/- 1.22 years) | Questionnaire | Health Promoting Lifestyle Profile - HPLP | Adolescents | Self | Usual dietary practices | No |
| Ghasab et al. 2019 (127) | Iran | RCT | 230 students (13 to 15 years) | Questionnaire | Global School-based Student Health Survey - GSSHS/ Youth Risk Behaviour Survey - YRBS | Adolescents | Interviewer | Usual dietary practices | No |
| Amos et al. 2012 (128) | Ghana | Cross-sectional | 150 students (18 to 20 years) | Questionnaire | Eating Habits Questionnaire for Adolescents - EHQA | Adolescents | Self | Eating habits / Factors influencing eating habits | Yes - Internal consistency |
| Gitau et al. 2014 (129) | South Africa | Cross-sectional | 340 students (13, 15, 17 years) | Questionnaire | Eating Attitudes Test - EAT | Adolescents | Interviewer | Eating attitudes | No |
| Maulida et al. 2016 (130) | Indonesia | Cross-sectional | 681 adolescents (13 to 14 years) | Questionnaire | Food choices questionnaire/ Nutritional knowledge | Adolescents | Self | Food choice motives | No |
| Sahingoz & Sanlier 2011 (131) | Turkey | Cross-sectional | 890 students (12 to 14 years) | Questionnaire | KIDMED | Adolescents | Self | Usual dietary practices | No |
| Steff et al. 2019 (132) | Romania | Cross-sectional | 153 students (10.8 +/- 3.5 years) | Questionnaire | Three-Factor Eating Questionnaire-R21 for children and adolescents - CTFEQ-R21 | Adolescents | Interviewer | Usual dietary practices | No |
| Loh et al. 2013 (133) | Malaysia | Cross-sectional | 554 students (13 years) | Questionnaire | Children’s Eating Behaviour Questionnaire - CEBQ | Adolescents | Self | Usual dietary practices | Yes - Face validity/ Internal consistency/ Test-retest reliability |
| Ndagire et al. 2019 (134) | Uganda | Cross-sectional | 621 students (8 to 19 years) | Questionnaire | Global Schools’ Health Survey - GSHS | Adolescents | Self | Food and vegetable intake | Yes - Test-retest reliability |
| Zahedi et al. 2014 (135) | Iran | Cross-sectional | 6640 students (6 to 18 years) | Questionnaire | Global School Health Survey - GSHS | Adolescents | Interviewer | Junk food consumption | Yes - Face validity/ content validity/ Test-retest reliability |
| Marchioni et al. 2007 (136) | Brazil | Cross-sectional | 49 students (16 to 19 years) | Questionnaire | Adolescent Food Frequency Questionnaire - AFFQ | Adolescents | Self | Usual dietary practices | Yes - Test-retest reliability |
| Ogunsile et al. 2016 (137) | Nigeria | Cross-sectional | 143 students (13.59 +/-1.49 years) | Questionnaire | Adolescents’ Knowledge of Healthy Eating Questionnaire - ADKHEQ / Adolescents’ Attitude to Healthy Eating Questionnaire - ADAHEQ | Adolescents | Self | Dietary knowledge, attitude, and practice | Yes - Face validity/ Content validity/ Internal consistency |
| Morales et al. 2014 (138) | Vanezuela | Cross-sectional | 200 students (15 to 19 years) | Questionnaire | Adolescent Food Habits Checklist - AFHC | Adolescents | Self | Food habits | Yes - Criterion validity/ Test-retest reliability |
| Whati, L. H. (139) | South Africa | Cross-sectional | 13 years | Questionnaire | Nutrition Knowledge Questionnaire | Adolescents | Self | Nutritional knowledge | Yes - Criterion validity/ Internal consistency |
| Vakili et al. 2013 (140) | Iran | Cross-sectional | 506 students (15 to 18 years) | Questionnaire | WHO & FAO one day diversity questionnaire | General population | Self | Dietary diversity | No |
| Neutzling et al. 2010 (141) | Brazil | Cross-sectional | 2209 students (13 to 14 years) | Questionnaire | Block Questionnaire | General population | Self | Usual dietary practices | Yes – Not specified |
| Correa et al. 2017 (142) | Brazil | Cross-sectional | 719 students (10 to 19 years) | Questionnaire | Food Consumption Markers Form - FCMF | General population | Interviewer | Usual food consumption | Yes – Not specified |
| Melaku et al. 2018 (143) | Ethiopia | Cross-sectional | 455 students (14 to 19 years) | Questionnaire | WHO and FAO 1-day diversity questionnaire | General population | Interviewer | Dietary diversity / Nutritional knowledge | Yes – Not specified |
| Li et al. 2010 (144) | China | Cross-sectional | 401 students (10 to 19 years) | Questionnaire | Not specified | Not specified | Self | Usual dietary practices | No |
| Majid et al. 2016 (145) | Malaysia | Cohort | 794 students (13 years) | Dietary Recall | 7-day diet history | Not specified | Self | Usual food consumption | No |
| Monika et al. 2012 (146) | India | Cross-sectional | 1814 students (12 to 18 years) | Questionnaire | Not specified (Adapted from HRIDAY-CATCH, Project EAT, and the SPAN survey) | Not specified | Self | Dietary intake/ Breakfast consumption | No |
| Olumakaiye et al. 2010 (147) | Nigeria | Cross-sectional | 120 students (10 to 19 years) | Questionnaire | Not specified - adapted from FANTA Household Dietary Diversity Score | Adolescents | Self | Dietary diversity | Yes - content validity, test-retest reliability |
| de AssumpÃ§Ã£o et al. 2012 (148) | Brazil | Cross-sectional | 409 adolescents (12 to 19 years) | Questionnaire | HEI - Healthy Eating Index | Not specified | Self | Usual dietary practices | Yes – Criterion validity (24hr dietary recall) / Test-retest reliability. |
| Jikamo & Samuel, 2019 (149) | Ethiopia | Cross-sectional | 2084 adolescents (13 to 17 years) | Questionnaire | Dietary diversity score | Not specified | Self | Usual dietary practices | No |
| Shaikh et al. 2017 (150) | India | Cross-sectional | 198 students (14 to 18 years) | Questionnaire | Nutrition Transition Food Frequency Questionnaire - NT-FFQ | Not specified | Interviewer | Usual dietary practices | Yes - content validity /Test-retest reliability |
| Ali and Abizari 2018 (151) | Ghana | Cross-sectional | 366 students (10 to 19 years) | Questionnaire / Dietary recall | Food Frequency Questionnaire (FFQ) / 24hr food recall | Not specified | Interviewer | Dietary diversity / Dietary patterns | No |
| Choeda et al 2021 (152) | India | Cross-sectional | 5809 students (13 to 17 years) | Questionnaire | Global School-Based Health Survey (GSHS) | Adolescents | Self | Dietary patterns | Not specified |
| Li et al 2020(153) | 60 LMIC countries * | Cross-sectional | 153496 students (12 to 15 years) | Questionnaire | Global School-Based Health Survey (GSHS) | Adolescents | Self | Fast food consumption | Yes |
| Pajuelo et al 2021(154) | Peru | Cross-sectional | 242 students (11 to 18 years) | Questionnaire | Not specified | Adolescents | Self | Knowledge, attitudes, and practices about healthy eating | Yes - Internal consistency |
| Slater et al. 2010 (155) | Brazil | Cohort | 95 students (5th and 8th grade) | Dietary Recall / Questionnaire | Food Frequency Questionnaire for Adolescents - FFQA / 24 hr recall | Not specified | Self | Usual dietary practices | Yes – Criterion validity (24hr dietary recall) |
| **Regional** | | | | | | | | | |
| Dalky et al. 2017 (156) | Jordan | Cross-sectional | 423 students (12 to 19 years) | Questionnaire | Arabic Eating Habits Questionnaire - AEHQ/ Eating Habits Questionnaire for Adolescents - EHQA | Adolescents | Self | Usual dietary practices | No |
| Ochoa-Aviles et al. 2014 (157) | Ecuadorian | Cross-sectional | 779 students (10 to 16 years) | Dietary recall | 24hr Dietary recall | Not specified | Interviewer | Usual dietary practices | No |
| **Country-specific** | | | | | | | | | |
| AllehdanSabika et al 2017 (158) | Jordan | Cross-sectional | 745 students (15 to 18 years) | Questionnaire | Not specified | Adolescents | Self | Perceptions about fast food | Yes - Test -retest reliability |
| Aounallah-Skhiri et al. 2011 (159) | Tunisia | Cross-sectional | 1019 adolescents (15 to 19 years) | Questionnaire | Food Frequency Questionnaire - FFQ | Adolescents | Self | Usual food consumption | Not specified |
| Azadbakht et al. 2015 (160) | Iran | Cross-sectional | 265 students (11 to 13 years) | Questionnaire | Food Frequency Questionnaire - FFQ | Adolescents | Self | Usual food consumption | Yes - Criterion validity (24hr dietary records) |
| Azeredo et al. 2016 (161) | Brazil | Cross-sectional | 109104 students (11 to 19 years) | Questionnaire | Food Frequency Questionnaire - FFQ | Adolescents | Self | Usual food consumption | Not specified |
| Borges et al 2012 (162) | Brazil | Cross-sectional | 1372 students (10 to 15 years) | Questionnaire | Semi Quantitative Food Frequency Questionnaire - SQFFQ | Adolescents | Self | Usual food consumption | Yes - Criterion validity (24hr dietary recalls) |
| Ahadi et al. 2015 (163) | Iran | Cross-sectional | 13486 students (6 to 18 years) | Questionnaire | Not specified | Adolescents | Self | Breakfast intake | No |
| Al Sabbah et al. 2007 (164) | West Bank and Gaza | Cross-sectional | 8885 students (12 to 18 years) | Questionnaire | Palestinian Health Behaviour in School-aged Children - HBSC | Adolescents | Self | Usual food consumption/ Breakfast during schooldays | No |
| Alavi et al. 2013 (165) | Iran | Cross-sectional | 386 students (13.2 years) | Questionnaire | Not specified | Adolescents | Self | Usual dietary practice / Nutrition knowledge / Attitudes towards nutrition | No |
| Barufaldi et al. 2016 (166) | Brazil | Cross-sectional | 74589 students (12 to 17 years) | Questionnaire | Not specified | Adolescents | Self | Usual dietary practices | No |
| Barufaldi et al. 2016 (167) | Brazil | Cross-sectional | 74589 students (12 to 17 years) | Questionnaire | Not specified | Adolescents | Self | Eating habits / Fluid intake/ Meals with parents | No |
| Belachew et al. 2013 (168) | Ethiopia | Cross-sectional | 2084 adolescents (13 to 17 years) | Questionnaire | Not specified | Adolescents | Interviewer | Usual dietary practice/ Food insecurity | No |
| Cacavas et al. 2011 (169) | Tonga | Cross-sectional | 2084 students (11 to 22 years) | Questionnaire | Not specified | Adolescents | Interviewer | Influence of the school environment on adolescent dietary habits | No |
| Cai-Xia et al. 2012 (170) | China | Cross-sectional | 2977 students (12 to 17 years) | Questionnaire | Not specified | Adolescents | Self | Fruit and vegetable consumption | No |
| Itani et al. 2017 (171) | Turkey | Cross-sectional | 482 students (17 to 18 years) | Questionnaire | Food Frequency Questionnaire - FFQ | Adolescents | Self | Usual food consumption | Yes - Content validity/ Construct validity/ Internal consistency/ Test-retest reliability |
| Joulaei et al. 2018 (172) | Iran | Cross-sectional | 420 students (13 to 15 years) | Questionnaire | Food Frequency Questionnaire - FFQ | Adolescents | Interviewer | Dietary intake / Nutritional knowledge/ Nutrition literacy | Yes - Criterion validity (24hr dietary records) |
| Feeley & Norris, 2014 (173) | South Africa | Cross-sectional | 1451 adolescents (17 to 18 years) | Questionnaire | Not specified | Adolescents | Self | Usual dietary practices | No |
| Feeley et al. 2012 (174) | South Africa | Cross-sectional | 1451 adolescents (17 to 18 years) | Questionnaire | Not specified | Adolescents | Interviewer | Dietary habits | No |
| Francis et al. 2009 (175) | Jamaica | Cross-sectional | 1317 adolescents (15 to 19 years) | Questionnaire | Not specified | Adolescents | Self | Fast food consumption / Alcohol consumption | No |
| Garba et al. 2014 (176) | Malaysia | Cross-sectional | 2248 students (13 to 17 years) | Questionnaire | Food Frequency Questionnaire - FFQ | Adolescents | Self | Usual food consumption | No |
| Korkalo et al. 2016 (177) | Mozambique | Cross-sectional | 551 adolescents (14 to 19 years) | Questionnaire | 7-day Food Frequency Questionnaire - FFQ | Adolescents | Self | Usual dietary practices | No |
| MacuÃ¡cua et al. 2019 (178) | Mozambique | Cross-sectional | 323 adolescents (10 to 14 years) | Questionnaire | Food Frequency Questionnaire - FFQ | Adolescents | Interviewer | Usual food consumption | No |
| Mikki et al. 2010 (179) | Palestine | Cross-sectional | 1000 students (13 to 15 years) | Questionnaire | Food Frequency Questionnaire - FFQ | Adolescents | Self | Usual food consumption | Yes - Test-retest reliability |
| Mirmiran et al. 2007 (180) | Iran | Cross-sectional | 7669 students (10 to 19 years) | Questionnaire | Food Frequency Questionnaire - FFQ | Adolescents | Self | Knowledge, attitudes, and practices on healthy nutrition / Usual food consumption | Yes - Criterion validity (24hr dietary records) |
| Mohamed et al. 2018 (181) | Malaysia | Cross-sectional | 78 students (13 to 15 years) | Questionnaire | MyUM Adolescent Food Frequency Questionnaire | Adolescents | Self | Usual dietary practices | Yes - Criterion validity (7-day dietary recall)/ Test-retest reliability |
| Musaiger & Kalam 2014 (182) | Syria | Cross-sectional | 365 students (15 to 18 years) | Questionnaire | Food Frequency Questionnaire - FFQ/ Dietary habits questionnaire | Adolescents | Self | Usual dietary practices | No |
| Ortiz-HernÃ¡ndez et al. 2008 (183) | Mexico | Cross-sectional | 7218 adolescents (12 to 19 years) | Questionnaire | 2005 National Youth Survey - NYS05 | Adolescents | Interviewer | Usual food consumption | Yes – Not specified |
| Patcheep 2015 (184) | Thailand | Cross-sectional | 148 students (15 to 18 years) | Questionnaire | Thai Eating Questionnaire - TEQ | Adolescents | Self | Eating intention and behaviour | Yes - Face validity/ content validity/ Internal consistency/ Test-retest reliability |
| Raghunatha Rao et al. 2007 (185) | India | RCT | 164 students (10th grade) | Questionnaire | Not specified | Adolescents | Interviewer | Usual dietary practices / Food preferences | No |
| Ranjana et al. 2013 (186) | Mauritius | Cross-sectional | 384 students (12 to 19 years) | Questionnaire | Not specified | Adolescents | Interviewer | Eating behaviour | No |
| Rodriguez et al. 2017 (187) | Peru | Cross-sectional | 118 adolescents (7.59 +/- 3.64 years) | Questionnaire | Food Frequency Questionnaire - FFQ | Adolescents | Interviewer | Usual food consumption | Yes - Test-retest reliability |
| Roba et al. 2015 (188) | Ethiopia | Cross-sectional | 188 adolescents (15 to 19 years) | Questionnaire | Food Frequency Questionnaire - FFQ | Adolescents | Interviewer | Usual dietary practices / Food insecurity | No |
| Saravia et al. 2018 (189) | South America | Cross-sectional | 357 adolescents (3 to 17 years) | Questionnaire | Food Frequency Questionnaire - FFQ | Adolescents | Self | Usual food consumption | No |
| Sedibe-Modiehi et al. 2018 (190) | South Africa | Cross-sectional | 3490 adolescents (11 to 15 years) | Questionnaire | Not specified | Adolescents | Interviewer | Usual dietary practices | Yes - Test-retest reliability |
| Seubsman et al. 2009 (191) | Thailand | Cross-sectional | 634 adolescents (15 to 19 years) | Questionnaire | Not specified | Adolescents | Self | Knowledge on risks of eating fast foods/ Fast-food consumption behaviour/ Perceptions on traditional foods | Yes - Face validity |
| Schneider et al. 2016 (192) | Brazil | Cross-sectional | 5249 adolescents (15 years) | Questionnaire | Food Frequency Questionnaire - FFQ | Adolescents | Interviewer | Usual food consumption | No |
| Soyer et al. 2008 (193) | Turkey | Cross-sectional | 527 students (15 years) | Questionnaire | Not specified | Adolescents | Self | Meal pattern / Food choice | No |
| Venter & Winterbach, 2010 (194) | South Africa | Cross-sectional | 168 students (17 years) | Questionnaire | Food Frequency Questionnaire - FFQ | Adolescents | Self | Dietary Fat Knowledge / Nutritional knowledge / Dietary fat intake | No |
| Wang et al. 2011 (195) | China | Cross-sectional | 3368 (11 to 18 years) | Questionnaire | Not specified | Adolescents | Self- | Dietary habits | No |
| Williams et al. 2019 (196) | Sri Lanka | Cross-sectional | 1300 students (12 to 18 years) | Questionnaire | Food Frequency Questionnaire - FFQ | Adolescents | Self | Usual food consumption/ Diet quality | No |
| Araujo et al. 2010 (197) | Brazil | Cross-sectional | 169 students (12 to 18 years) | Questionnaire | Food Frequency Questionnaire - FFQ | General population | Self | Usual food consumption | Yes - Criterion validity (3 24hr dietary records) |
| Abudayya et al. 2009 (198) | Gaza | Cross-sectional | 944 students (12 to 15 years) | Questionnaire | Food Frequency Questionnaire - FFQ | General population | Self | Usual food consumption | No |
| Areekul et al. 2005 (199) | Thailand | Cross-sectional | 298 students (12 to 15 years) | Questionnaire | Food Frequency Questionnaire - FFQ | General population | Self | Usual food consumption | No |
| Chiarelli et al 2011 (200) | Brazil | Cross-sectional | 268 students (12 to 16 years) | Questionnaire | Food Frequency Questionnaire - FFQ | General population | Self | Usual food consumption | No |
| Henn et al. 2010 (201) | Brazil | Cross-sectional | 268 students (12 to 19 years) | Questionnaire | Food Frequency Questionnaire - FFQ-Porto Alegre/ 24 hr dietary recall | General population | Self | Usual dietary practices | Yes - Criterion validity (2 24hr dietary records) |
| Niu et al. 2014 (202) | China | Longitudinal | 3923 adolescents (6 to 17 years) | Questionnaire | China Health and Nutrition Survey - CHNS | General population | Interviewer | Weight perception/ Nutrition knowledge | No |
| RodrÃ­guez et al. 2017 (203) | Mexico | Cross-sectional | 2203 adolescents (12 to 19 years) | Questionnaire | Food Frequency Questionnaire - FFQ | General population | Self | Usual food consumption | No |
| Singh et al. 2019 (204) | Nepal | Cross-sectional | 407 adolescents (10 to 19 years) | Questionnaire | Food Frequency Questionnaire - FFQ | General population | Interviewer | Fruit and vegetable consumption | No |
| Wate et al. 2013 (205) | Fiji | Cross-sectional | 6871 students (13 to 18 years) | Questionnaire | Food Frequency Questionnaire - FFQ | General population | Self | Usual food consumption | No |
| Abizari & Ali, 2019 (206) | Ghana | Cross-sectional | 366 students (10 to 19 years) | Dietary recall | 24hr Dietary recall | Not specified | Self | Usual dietary practices/ Dietary diversity | No |
| Bakir et al. 2015 (207) | Turkey | Cross-sectional | 598 students (11 to 17 years) | Dietary Recall | 24hr Dietary recall | Not specified | Interviewer | Usual food consumption | No |
| Bardosono et al. 2015 (208) | Indonesia | Repeat Cross-sectional | 303 adolescents (16 to 18 years) | Questionnaire | 24hr dietary recall / 7-day fluid intake | Not specified | Interviewer | Food and fluid intake | No |
| Birru et al. 2018 (209) | Ethiopia | Cross-sectional | 778 students (15.49 +/- 1.93 years) | Dietary recall | 24hr Dietary recall | Not specified | Interviewer | Dietary diversity | No |
| Borges et al. 2018 (210) | Brazil | Cross-sectional | 6784 adolescents (10 to 18 years) | Dietary Record | 24hr Food record | Not specified | Self | Usual dietary practices | No |
| Bullecer et al. 2012 (211) | Philippines | Cross-sectional | 571 students (16 to 19 years) | Dietary Record | 24hr Food record | Not specified | Self | Usual dietary practices | No |
| Cunha et al. 2018 (212) | Brazil | Cross-sectional | 7613 adolescents (10 to 19 years) | Dietary Record | 2-day food record | Not specified | Self | Usual dietary practices | No |
| da Costa Louzada et al. 2015 (213) | Brazil | Cross-sectional | 3760 adolescents (>10 years) | Dietary Record | 24hr Food record | Not specified | Self | Usual dietary practices | No |
| Das et al. 2016 (214) | India | Cross-sectional | 150 students (11 to 19 years) | Dietary Record | 24hr Dietary record | Not specified | Interviewer | Usual dietary practices | No |
| de Andrade et al. 2010 (215) | Brazil | Cross-sectional | 1584 adolescents (12 to 20 years) | Dietary Recall | 24hr Dietary recall | Not specified | Interviewer | Usual dietary practices / Diet quality | No |
| de Morais et al. 2013 (216) | Brazil | Cross-sectional | 430 students (10 to 19 years) | Dietary recall | 24hr Dietary recall | Not specified | Self | Usual dietary practices | No |
| Dixit et al. 2014 (217) | India | Cross-sectional | 576 adolescents (10 to 19 years) | Dietary Recall | 24hr Dietary recall | Not specified | Self | Usual dietary practices | No |
| Esmaillzadeh et al. 2012 (218) | Iran | Cross-sectional | 257 students (11 to 15 years) | Questionnaire | Food Frequency Questionnaire - FFQ | Not specified | Self | Usual food consumption | No |
| Estrada-Reyes et al. 2018 (219) | Mexico | Cross-sectional | 56 students (14 to 17 years) | Dietary Record | 24hr Food record | Not specified | Self | Usual dietary practices | No |
| Funke et al. 2007 (220) | Nigeria | Cross-sectional | 401 students (10 to 19 years) | Questionnaire | Food Frequency Questionnaire - FFQ | Not specified | Interviewer | Usual food consumption | No |
| Garipagaoglu et al. 2008 (221) | Turkey | Cross-sectional | 1944 students (12 to 17 years) | Dietary Record | 3-day food record | Not specified | Self | Usual dietary practices | No |
| Korkalo et al. 2014 (222) | Mozambique | Cross-sectional | 551 adolescents (14 to 19 years) | Dietary Recall / Questionnaire | Food Frequency Questionnaire - FFQ/ 24hr dietary recall | Not specified | Interviewer | Usual dietary practices | No |
| Mascarenhas et al. 2016 (223) | Brazil | Cross-sectional | 70 students (11 to 17 years) | Dietary Record | 24hr Food record | Not specified | Self | Usual dietary practices | Yes - Criterion validity (FFQ) |
| Magbuhat et al. 2011 (224) | Philippines | Cross-sectional | 120 students (13 to 17 years) | Dietary Record | 3-day food record / Food preference checklist | Not specified | Self | Dietary intake / Food preference | No |
| Majid et al. 2016 (225) | Malaysia | Cross-sectional | 820 students (13 years) | Dietary recall | 7-day diet history | Not specified | Interviewer | Usual dietary practices | No |
| Mascarenhas et al. 2014 (226) | Brazil | Cross-sectional | 1330 students (11 to 17 years) | Questionnaire | Food Frequency Questionnaire - FFQ | Not specified | Interviewer | Usual food consumption/ Diet quality | No |
| Mirmiran et al. 2004 (227) | Iran | Cross-sectional | 304 (10 to 18 years) | Dietary Recall | 24hr Dietary recall | Not specified | Self | Usual food consumption | No |
| Nabhani-Zeidan et al. 2011 (228) | Lebanon | Cross-sectional | 320 students (17 to 19 years) | Dietary Recall / Questionnaire | 24hr Dietary recall/ Not specified | Not specified | Self | Nutritional knowledge / Usual dietary practices | Yes - Test-retest reliability |
| Monge-Rojas 2001 (229) | Costa Rica | Cross-sectional | 328 students (12 to 19 years) | Dietary Record | 3-day food record | Not specified | Interviewer | Fast food consumption / Perception of fast food / Location of fast-food places | No |
| Napier & Hlambelo 2014 (230) | South Africa | Cross-sectional | 61 students (13 to 18 years) | Questionnaire / Dietary Record | 24hr Dietary recall / Weighed food record | Not specified | Interviewer | Dietary intake / Lunchbox content | No |
| Neto et al. 2015 (231) | Brazil | Cross-sectional | 2994 students (10 to 19 years) | Questionnaire | Food Frequency Questionnaire - FFQ | Not specified | Interviewer | Usual food consumption | No |
| Nithya & Bhavani, 2018 (232) | India | Cross-sectional | 183 adolescents (13 to 17 years) | Dietary Recall / Questionnaire | 24hr Dietary recall /Food Frequency Questionnaire - FFQ | Not specified | Self | Usual dietary practices / Dietary diversity | No |
| Olumakaiye et al. 2007(233) | Nigeria | Cross-sectional | 401 students (10 to 19 years) | Questionnaire | Not specified | Not specified | Self | Usual dietary practices | Yes - Content validity / test-retest reliability |
| Ogunkunle et al. 2013 (234) | Nigeria | Cross-sectional | 302 students (10 to 19 years) | Dietary Recall / Questionnaire | 24hr Dietary recall /Food Frequency Questionnaire - FFQ | Not specified | Interviewer | Usual dietary practices | No |
| Oldewage-Theron et al. 2015 (235) | South Africa | Cross-sectional | 98 students (16 +/- 1.4 years) | Dietary Recall / Questionnaire | 24hr Dietary recall /Food Frequency Questionnaire - FFQ | Not specified | Interviewer | Usual dietary practices | No |
| Onyiriuka et al. 2013 (236) | Nigeria | Cross-sectional | 2097 students (12 to 19 years) | Questionnaire | Not specified | Not specified | Self | Usual dietary practices | No |
| Onyiriuka et al. 2013 (237) | Nigeria | Cross-sectional | 2097 students (12 to 19 years) | Questionnaire | Food Frequency Questionnaire - FFQ | Not specified | Self | Usual dietary practices / Food choices influence | No |
| Oogarah-Pratap 2007 (238) | Mauritius | Cross-sectional | 315 students (13 to 16 years) | Questionnaire | Food Frequency Questionnaire - FFQ | Not specified | Self | Dietary practices/ Sources of nutrition information /Types of dietary advice given to friends and relatives. | No |
| Otuneye et al. 2017 (239) | Nigeria | Cross-sectional | 1550 students (10 to 19 years) | Questionnaire | Not specified | Not specified | Interviewer | Usual dietary practices / Diet quality | No |
| Pangan et al. 2012 (240) | Philippines | Cross-sectional | 99 students (16 to 21 years) | Dietary Record | 24hr Dietary record | Not specified | Self | Fast food consumption | No |
| Rezali et al. 2015 (241) | Malaysia | Cross-sectional | 373 students (13 to 16 years) | Dietary Recall | 24hr Dietary recall | Not specified | Self | Usual food consumption / Diet quality/ Healthy food availability at home | No |
| Rouhani et al. 2012 (242) | Iran | Cross-sectional | 140 students (11 to 13 years) | Questionnaire | Food Frequency Questionnaire - FFQ | Not specified | Self | Usual food consumption | Yes - Criterion validity (3 24hr dietary records) |
| Ronca et al. 2019 (243) | Brazil | Cross-sectional | 71553 students (12 to 17 years) | Dietary Recall | 24hr Dietary recall | Not specified | Interviewer | Usual food consumption | No |
| Samaranayaka et al. 2013 (244) | Sri Lanka | Cross-sectional | 600 students (14.6 +/-2.3 years) | Questionnaire | Food Frequency Questionnaire - FFQ | Not specified | Self | Usual food consumption | No |
| Som et al. 2016 (245) | India | Cross-sectional | 506 adolescents (14 to 19 years) | Questionnaire | Food Frequency Questionnaire - FFQ | Not specified | Self | Usual food consumption / Weight concerns | No |
| Tayel et al. 2013 (246) | Egypt | Cross-sectional | 300 students (12 to 18 years) | Questionnaire | Food Frequency Questionnaire - FFQ | Not specified | Interviewer | Usual food consumption/ Nutritional knowledge | No |
| Tehrani et al. 2019 (247) | Iran | Cross-sectional | 297 students (15 to 18 years) | Questionnaire | Food Frequency Questionnaire - FFQ | Not specified | Self | Usual food consumption/ Diet quality | Yes - Criterion validity (12 24hr dietary records), |
| Tek et al. 2011 (248) | Turkey | Cross-sectional | 1104 students (14 to 18 years) | Dietary recall | 24hr Dietary recall | Not specified | Interviewer | Usual dietary practices | No |
| Wiecha et al. 1994 (249) | Vietnam | Cross-sectional | 35 students (13 to 23 years) | Questionnaire | Food Frequency Questionnaire - FFQ | Not specified | Self | Usual food consumption | No |
| Wei et al. 2011 (250) | China | Cross-sectional | 168 students (12 to 18 years) | Questionnaire | Food Frequency Questionnaire - FFQ/ 24hr dietary recall | Not specified | Interviewer | Usual dietary practices | Yes - Criterion validity (3 24hr dietary records) |
|  |  |  |  |  |  |  |  |  |  |
| El-Gilany and Elkhawaga 2012 (251) | Egypt | Cross-sectional | 891 students (14 to 20 years) | Questionnaire | Not specified | Not specified | Self | Meal patterns | No |
| Ahmed 1998 (252) | Bangladesh | Cross-sectional | 384 students (10 to 16 years) | Questionnaire/Dietary recall | 7-day Food Frequency Questionnaire (FFQ) / 24hr recall | Not specified | Self | Food consumption patterns | No |
| Kalkan et al 2015 (253) | Turkey | Cross-sectional | 643 students (13-16 years) | Questionnaire | Adolescent Food Habits Checklist (AFHC) | Adolescents | Self | Food consumption patterns | No |
| Alam 2010 (254) | Bangladesh | Cross-sectional | 4993 students (13 to 18 years) | Questionnaire | Food checklist | Not specified | Interviewer | Dietary knowledge / Food consumption frequency | No |
| Alangea 2018 (255) | Ghana | Cross-sectional | 487 adolescents (9 to 15 years) | Questionnaire | Food Frequency Questionnaire (FFQ) | Adolescent | Self | Food consumption patterns | No |
| Amos et al. 2012 (256) | Ghana | Cross-sectional | 150 students (12 to 20 years) | Questionnaire | Eating Habits Questionnaire for Adolescents (EHQA) | Adolescent | Self | Eating Habits | Yes - Reliability |
| Aounallah-Skhiri 2008 (257) | Tunisia | Cross-sectional | 2872 adolescents (15 to 19 years) | Questionnaire | Food Frequency Questionnaire (FFQ) | Not specified | Self | Eating habits | No |
| Araujo et al 2008 (258) | Brazil | Cross-sectional | 114 adolescents (11 to 19 years) | Questionnaire | Semi-quantitative Food Frequency Questionnaire (FFQ) (QSFA) | Not specified | Interviewer | Usual dietary consumption | Yes - Intraclass corelation |
| Aroa et al 2012 (259) | India | Cross-sectional | 1814 students (12 to 18 years) | Questionnaire | Behavioral-psychosocial (BP) survey | Not specified | Self | Breakfast consumption | Not specified |
| Azeredo et al. 2014 (260) | Brail | Cross-sectional | 109104 students (11 to 14 years) | Questionnaire | National Survey of Schoolchildren’s Health (2012) | Adolescent | Self | Dietary consumption | Not specified |
| Carvalho et al. 2001 (261) | Brazil | Cross-sectional | 335 students (10 to 13 years) | Questionnaire | Food Frequency Questionnaire (FFQ) | Not specified | Self | Frequency of food consumption | No |
| Estima et al. 2009 (262) | Brazil | Cross-sectional | 528 (12 to 18 years) | Questionnaire | Food Frequency Questionnaire (FFQ) | Not specified | Self | Meal patterns | No |
| Ilesanmi 2014 (263) | Nigeria | Cross-sectional | 294 students (12 to 19 years) | Questionnaire | Not specified | Not specified | Self | Fruit consumption | No |
| letlape et al. 2011 (264) | South Africa | Cross-sectional | 500 students (15 to 18 years) | Questionnaire | Not specified | Adolescent | self | Nutrition knowledge | Not specified |
| Lopez et al 2012 (265) | Morocco | Cross-sectional | 327 students (15 to 20 years) | Dietary recall | 24hr Dietary recall | General population | Self | Food consumption | Not specified |
| Monge-Rojas et al 2013 (266) | Costa Rica | Cross-sectional | 400 adolescents (12 to 17 years) | Questionnaire | Not specified | General population | Self | Fast food consumption | No |
| Montazerifar et al 2012 (267) | Iran | Cross-sectional | 753 girls (14 to 18 years) | Questionnaire / Dietary recall | Food Frequency Questionnaire (FFQ)/ 24hr Dietary recall | Not specified | Self | Dietary intake / Food habits | No |
| Naja 2015(268) | Lebanon | Cross-sectional | 446 adolescents (13 to 19 years) | Questionnaire | Food Frequency Questionnaire (FFQ) | Not specified | Self | Dietary intake | No |
| Nurul-Fadhilah et al 2012 (269) | Malaysia | Cross-sectional | 170 adolescents | Questionnaire | Food Frequency Questionnaire (FFQ) | Adolescent | Self | Food consumption | Yes - Criterion validity (3-day 24hr recall) |
| Nurul-Fadhilah et al 2013(270) | Malaysia | Cross-sectional | 236 adolescents (12 to 19 years) | Questionnaire | Food Frequency Questionnaire (FFQ) | Adolescent | Self | Breakfast consumption | Yes - Criterion validity (3-day 24hr recall) |
| Peltzer 2012 (271) | Southeast Asia countries (India, Indonesia, Myanmar, Sri Lanka and Thailand) | Cross-sectional | 16084 students (13 to 15 years) | Questionnaire | Global School-Based Health Survey (GSHS) | Adolescent | Self | Fruit and vegetable consumption | No |
| Peltzer 2016 (272) | Southeast Asia countries (India, Indonesia, Myanmar, Sri Lanka and Thailand) | Cross-sectional | 30284 students (13 to 15 years) | Questionnaire | Global School-Based Health Survey (GSHS) | Adolescent | Self | Habitual physical activity | No |
| Sakar 2015 (273) | India | Cross-sectional | 150 adolescents (10 to 19 years) | Questionnaire | Not named | Adolescent | Self | Eating patterns and habits | No |
| Shi et al 2005 (274) | China | Cross-sectional | 824 students (12 to 14 years) | Questionnaire | Food Frequency Questionnaire (FFQ) | General population | Self | Meal frequency / Food frequency / Food preferences | Yes - Test-retest reliability |
| Slater et al 2003 (275) | Brazil | Cross-sectional | 79 adolescents (14 to 18 years) | Questionnaire | Food Frequency Questionnaire for Adolescents (FFQ-A) | General population | Self | Food consumption | Yes - Criterion validity (3-day 24hr recall) |
| Xia et al 2011 (276) | China | Cross-sectional | 168 adolescents (12 to 18 years) | Questionnaire | Food Frequency Questionnaire (FFQ) | Adolescent | Self | Food consumption | Yes - Criterion validity (3-day 24hr recall) |
| Zhang et al 2012 (277) | China | Cross-sectional | 2977 students (12 to 17 years) | Questionnaire | Semi-quantitative Food Frequency Questionnaire (FFQ) | General population | Self | Food consumption | Yes - Test-retest reliability |
| Zanini et al 2013 (278) | Brazil | Cross-sectional | 600 students (15 to 20 years) | Questionnaire | Risk behaviors in adolescents from Santa Catarina (COMCAP) | Adolescent | Self | Daily food consumption | Not specified |
| Okeyo et al 2020 (279) | South Africa | Cross-sectional | 1360 students (11 to 26 years) | Questionnaire | Food Frequency Questionnaire (FFQ) | Adolescents | Self | Dietary practices | No |
| Neta et al 2021 (280) | Brazil | Longitudinal | 1431, 1178, 959, and 773 students (from 2012 to 2017) (10 to 14 years in 2014) | Dietary recall | 24hr diet recall | Not specified | Self | Food consumption / dietary patterns | Not specified |
| Fatikhani and Setiwan 2019 (281) | Indonesia | Cross-sectional | 190 students (15 to 16 years) | Questionnaire | Not named and Sari dietary instrument | Not specified | Self | Knowledge about fast food / dietary habits | Yes - Reliability |
| Mohammmadbeigi et al 2019 (282) | Sri Lanka | Cross-sectional | 638 students (15 to 18 years) | Questionnaire | Food Frequency Questionnaire (FFQ) | Adolescents | Self | Junk food consumption | Yes - Reliability |
| Said et al 2020 (283) | Lebanon | Cross-sectional | 1535 students (15 to 18 years) | Questionnaire | Dietary Knowledge Questionnaire (DKQ) / Dietary Adherence Questionnaire (DAQ) | Adolescents | Interviewer | Dietary knowledge / Dietary adherence | Yes - Internal consistency |
| Said et al 2020 (284) | Lebanon | Cross-sectional (validation study) | 220 students (15 to 18 years) | Questionnaire | Dietary Knowledge Questionnaire (DKQ) / Dietary Adherence Questionnaire (DAQ) | Adolescents | Interviewer | Dietary knowledge / Dietary adherence | Yes - Criterion validity (24hr diet recall) / Internal consistency |
| Tunkara-Bah 2021 (285) | The Gambia | Cross-sectional | 1008 students (13 to 19 years) | Questionnaire | Not specified | Adolescents | Self | Dietary habits | No |
| Giguère-Johnson et al 2021 (286) | Senegal | Cross-sectional | 136 students (14 to 18 years) | Dietary recall | 3-day 24hr diet recalls | Not specified | Interviewer | Food intake / Eating behaviour (breakfast, three meals daily, meals outside of home, fruit, and vegetable consumption) | No |
| Abizari et al 2019 (287) | Ghana | Cross-sectional | 366 students (10 to 19 years) | Questionnaire | Food Frequency Questionnaire (FFQ) | Not specified | Self | Food consumption / Dietary patterns | No |
| Bezerra et al 2020 (288) | Brazil | Cross-sectional | 71740 adolescents (12 to 17 years) | Dietary recall | 24hr food recall | Adolescents | Interviewer | Away-from-home food consumption | No |
| Agofure et al. 2021 (289) | Nigeria | Cross-sectional | 129 students (16 to 18 years) | Questionnaire | Not specified | Not specified | Self | Eating patterns and habits | Yes - Internal consistency / Face validity (field experts) |
| Thorne - Lyman et al 2020 (290) | Bangladesh | Cross-sectional | 30702 adolescents (9 to 15 years) | Questionnaire | 7-day Food Frequency Questionnaire (FFQ) | Not specified | Self | Dietary patterns | Yes - Criterion validity |
| Teixeira et al 2021 (291) | Brazil | Cross-sectional | 720 adolescents (10 to 18 years) | Questionnaire | National Adolescent School-based Health Survey (PeNSE) | Adolescents | Self | Dietary patterns (food consumption, meal patterns) | Yes - Validity and Reliability |
| Korkalo et al. 2012 (292) | Mozambique | Cross-sectional | 100 adolescents (13 to 18 years) | Dietary Recall / Observational | Dietary recall/ Weighted dietary intake observation | Not specified | Observation | Usual dietary practices | No |
|  |  |  |  |  |  |  |  |  |  |
|  |  |  |  |  |  |  |  |  |  |

**REFERENCES**

1. Christofaro DGD, De Andrade SM, Mesas AE, Fernandes RA, Farias Júnior JC. Higher screen time is associated with overweight, poor dietary habits and physical inactivity in Brazilian adolescents, mainly among girls. European Journal of Sport Science. 2016;16(4):498-506.

2. Farah Wahida Z, Mohd Nasir MT, Hazizi AS. Physical Activity, Eating Behaviour and Body Image Perception among Young Adolescents in Kuantan, Pahang, Malaysia. Malaysian Journal of Nutrition. 2011;17(3):325-36.

3. Hatami M, Mohd. Taib MN, Jamaluddin R, Saad HA, Djazayery A, Chamari M, et al. Dietary factors as the major determinants of overweight and obesity among Iranian adolescents A cross-sectional study. Appetite. 2014;82:194-201.

4. Kafeshani O, Sarrafzadegan N, Nouri F, Mohammadifard N. Major dietary patterns in Iranian adolescents: Isfahan Healthy Heart Program, Iran. 2015. p. 61-8.

5. Masoomi H, Taheri M, Irandoust K, H’Mida C, Chtourou H. The relationship of breakfast and snack foods with cognitive and academic performance and physical activity levels of adolescent students. Biological Rhythm Research. 2019.

6. Safiri S, Kelishadi R, Qorbani M, Lotfi R, Djalalinia S, Salehifar D, et al. Association of dietary behaviors with physical activity in a nationally representative sample of children and adolescents: The CASPIAN- IV study. International Journal of Pediatrics. 2016;4(3):1505-17.

7. Saikia D, Ahmed SJ, Saikia H, Sarma R. Overweight and obesity in early adolescents and its relation to dietary habit and physical activity: A study in Dibrugarh town. Clinical Epidemiology and Global Health. 2016;4:S22-S8.

8. Prioreschi A, Wrottesley SV, Cohen E, Reddy A, Said-Mohamed R, Twine R, et al. Examining the relationships between body image, eating attitudes, BMI, and physical activity in rural and urban South African young adult females using structural equation modeling. PLoS One. 2017;12(11).

9. McArthur LH, Holbert D, Peña M. Development and application of rapid assessment diet and physical activity indexes, which suggest high consumption of energy-dense foods and inadequate exercise among adolescents from 6 Latin American cities: a pilot study. Nutrition Research. 2008;28(9):590-9.

10. Smith BJ, Phongsavan P, Havea D, Halavatau V, Chey T. Body mass index, physical activity and dietary behaviours among adolescents in the Kingdom of Tonga. Public Health Nutrition. 2007;10(2):137-44.

11. Tayyem RF, Albataineh SR, Allehdan S, Badran E. Relative validity and reproducibility of a food frequency questionnaire to evaluate food group intakes among jordanian children and adolescents. Nutricion Hospitalaria. 2021;38(5):1047-57.

12. Musaiger AO, Al-Mufty BA, Al-Hazzaa HM. Eating habits, inactivity, and sedentary behavior among adolescents in Iraq: Sex differences in the hidden risks of noncommunicable diseases. Food and Nutrition Bulletin. 2014;35(1):12-9.

13. Çitozi R, Bozo D, Pano G. An assessment of the perception of physical activity, eating habits, self-efficacy and the knowledge about healthy food in Albanian adolescents. Journal of Human Sport and Exercise. 2013;8(2 SUPPL):192-203.

14. Abdullah NF, Teo PS, Foo LH. Ethnic differences in the food intake patterns and its associated factors of adolescents in Kelantan, Malaysia. Nutrients. 2016;8(9).

15. Chansukree P, Rungjindarat N. Social Cognitive Determinants of Healthy Eating Behaviors in Late Adolescents: A Gender Perspective. Journal of Nutrition Education & Behavior. 2017;49(3):204-10.e1.

16. Flor-Garrido P, Romo ML, Abril-Ulloa V. Differences in nutritional status, physical activity, and fruit and vegetable consumption in urban and rural school-going adolescents in Paute, Ecuador. Archivos Latinoamericanos De Nutricion. 2016;656(3):230-8.

17. Kelishadi R, Qorbani M, Motlagh ME, Ardalan G, Heshmat R, Hovsepian S. Socioeconomic disparities in dietary and physical activity habits of Iranian children and adolescents: The CASPIAN-IV study. Archives of Iranian medicine. 2016;19(8):530-7.

18. Musaiger AO, Al-Mannai M, Tayyem R, Al-Lalla O, Ali EY, Kalam F, et al. Risk of disordered eating attitudes among adolescents in seven Arab countries by gender and obesity: a cross-cultural study. Appetite. 2013;60:162-7.

19. Trang NHHD, Hong TK, Dibley MJ. Cohort profile: Ho Chi Minh City Youth cohort-changes in diet, physical activity, sedentary behaviour and relationship with overweight/obesity in adolescents. BMJ Open. 2012;2(1).

20. Musaiger AO, Nabag FO, Al-Mannai M. Obesity, Dietary Habits, and Sedentary Behaviors among Adolescents in Sudan. Food and Nutrition Bulletin. 2016;37(1):65-72.

21. De Oliveira LMFT, Da Silva AO, Dos Santos MAM, Ritti‑Dias RM, Diniz PRB. Exercise or physical activity: Which is more strongly associated with the perception of sleep quality by adolescents? Rev Paul Pediatr. 2018;36(3):322-8.

22. da Costa BGG, Chaput J-P, Lopes MVV, Malheiros LEA, Silva KSd. Associations between sociodemographic, dietary, and substance use factors with self-reported 24-hour movement behaviors in a sample of Brazilian adolescents. International Journal of Environmental Research and Public Health. 2021;18(5):2527.

23. Zalilah M, Khor G, Mirnalini K, Norimah A, Ang M. Dietary intake, physical activity and energy expenditure of Malaysian adolescents. Singapore medical journal. 2006;47(6):491.

24. Djordjevic-Nikic M, Dopsaj M. Characteristics of eating habits and physical activity in relation to body mass index among adolescents. Journal of the American College of Nutrition. 2013;32(4):224-33.

25. Asare M, Danquah SA. The relationship between physical activity, sedentary behaviour and mental health in Ghanaian adolescents. Child and Adolescent Psychiatry and Mental Health. 2015;9(1).

26. Azizi-Soleiman F, Motlagh ME, Qorbani M, Heshmat R, Ardalan G, Mansourian M, et al. Dietary habits and health related behaviors in Iranian children and adolescents: The CASPIAN- IV study. International Journal of Pediatrics. 2016;4(7):2087-97.

27. Chen ST, Liu Y, Hong JT, Tang Y, Cao ZB, Zhuang J, et al. Co-existence of physical activity and sedentary behavior among children and adolescents in Shanghai, China: Do gender and age matter? BMC Public Health. 2018;18(1).

28. D'Alonzo KT, Cortese LB. An Investigation of Habitual and Incidental Physical Activity Among Costa Rican and Costa Rican American Teenage Girls. Journal of Transcultural Nursing. 2007;18(3):201-7.

29. Dan SP, Mohd. Nasir MT, Zalilah MS. Sex and ethnic differentials in physical activity levels of adolescents in Kuantan. Malaysian Journal of Nutrition. 2007;13(2):109-20.

30. Dave H, Nimbalkar SM, Vasa R, Phatak AG. Assessment of physical activity among adolescents: A cross-sectional study. Journal of Clinical and Diagnostic Research. 2017;11(11):SC21-SC4.

31. de Fátima Guimarães R, da Silva MP, Legnani E, Mazzardo O, de Campos W. Reproducibility of adolescent sedentary activity questionnaire (ASAQ) in Brazilian adolescents. Revista Brasileira de Cineantropometria e Desempenho Humano. 2013;15(3):276-85.

32. Fazah A, Jacob C, Moussa E, El-Hage R, Youssef H, Delamarche P. Activity, inactivity and quality of life among Lebanese adolescents. Pediatrics International. 2010;52(4):573-8.

33. Glozah FN, Pevalin DJ. Perceived social support and parental education as determinants of adolescents' physical activity and eating behaviour: A cross-sectional survey. Int J Adolesc Med Health. 2015;27(3):253-9.

34. Greca JPDA, Silva DAS, Loch MR. Physical activity and screen time in children and adolescents in a medium size town in the South of Brazil. Rev Paul Pediatr. 2016;34(3):316-22.

35. Jabeen I, Zuberi R, Nanji K. Physical activity levels and their correlates among secondary school adolescents in a township of Karachi, Pakistan. Journal of the Pakistan Medical Association. 2018;68(5):737-43.

36. Kundapur R, Baisil S. Assessment of difference in physical activities in urban and rural adolescents of Mangalore. Indian Journal of Community Health. 2017;29(1):75-80.

37. Lachat CK, Verstraeten R, Hagströmer M, Khan NC, Van NDA, Dung NQ, et al. Validity of two physical activity questionnaires (IPAQ and PAQA) for Vietnamese adolescents in rural and urban areas. International Journal of Behavioral Nutrition and Physical Activity. 2008;5(1):37.

38. Lennox A, Pienaar AE, Wilders C. Physical fitness and the physical activity status of 15-year-old adolescents in a semi-urban community. South African Journal for Research in Sport, Physical Education and Recreation. 2008;30(1):59-73.

39. Li M, Dibley MJ, Sibbritt DW, Zhou X, Yan H. Physical activity and sedentary behavior in adolescents in Xi'an City, China. Journal of Adolescent Health. 2007;41(1):99-101.

40. Mamabolo RL, Kruger HS, Lennox A, Monyeki MA, Pienaar AE, Underhay C, et al. Habitual physical activity and body composition of black township adolescents residing in the North West Province, South Africa. Public Health Nutrition. 2007;10(10):1047-56.

41. Motamed-Gorji N, Qorbani M, Nikkho F, Asadi M, Motlagh ME, Safari O, et al. Association of screen time and physical activity with health-related quality of life in Iranian children and adolescents. Health & Quality of Life Outcomes. 2019;17(1):N.PAG-N.PAG.

42. Oyeyemi AL, Ishaku CM, Deforche B, Oyeyemi AY, De Bourdeaudhuij I, Van Dyck D. Perception of built environmental factors and physical activity among adolescents in Nigeria. International Journal of Behavioral Nutrition & Physical Activity. 2014;11:1-20.

43. Oyeyemi AL, Ishaku CM, Oyekola J, Wakawa HD, Lawan A, Yakubu S, et al. Patterns and Associated Factors of Physical Activity among Adolescents in Nigeria. Plos One. 2016;11(2).

44. Ramezankhani A, Tavassoli E, Ghafari M, Alidosti M, Daniali SS, Gharlipour Z. Physical activity in adolescent girls and their perceptions of obesity prevention in Shahr-e Kord, Iran. International Journal of Pediatrics. 2016;4(8):3249-62.

45. Sanaeinasab H, Saffari M, Nazeri M, Karimi Zarchi A, Cardinal BJ. Descriptive analysis of Iranian adolescents' stages of change for physical activity behavior. Nursing & Health Sciences. 2013;15(3):280-5.

46. Shokrvash B, Majlessi F, Montazeri A, Nedjat S, Rahimi A, Djazayeri A, et al. Correlates of physical activity in adolescence: A study from a developing country. Global Health Action. 2013;6(1).

47. Taymoori P, Berry TR, Lubans DR. Tracking of physical activity during middle school transition in Iranian adolescents. Health Education Journal. 2012;71(6):631-41.

48. Abasi MH, Eslami AA, Rakhshani F. Introducing an outcome expectation questionnaire and its psychometric properties regarding leisure time physical activity for Iranian male adolescent. Iran Red Crescent MedJ. 2015;17(5).

49. Fortes LS, Morgado FFR, Almeida SS, Ferreira MEC. Eating behavior and physical activity in adolescents. Rev Nutr. 2013;26(5):529-37.

50. Iskandar MM, Mohamad N, Othman S. Physical activity and BMI level: Youth in low-cost housing Kuala Lumpur. Pertanika Journal of Social Sciences and Humanities. 2017;25(June):43-53.

51. Kelishadi R, Ardalan G, Gheiratmand R, Gouya MM, Razaghi EM, Delavari A, et al. Association of physical activity and dietary behaviours in relation to the body mass index in a national sample of Iranian children and adolescents: CASPIAN Study. Asociación de la actividad física y los hábitos alimentarios en relación con el índice de masa corporal en una muestra nacional de niños y adolescentes iraníes: estudio CASPIAN. 2007;85(1):19-26.

52. Masocha V, Czyż SH, Moss SJ, Monyeki AM. Two-year changes in body composition, physical activity and selected metabolic risk factors among adolescents living in tlokwe municipality area, North West Province, South Africa: The PAHL study. South African Journal for Research in Sport, Physical Education and Recreation. 2018;40(2):99-114.

53. Paudel S, Subedi N, Bhandari R, Bastola R, Niroula R, Poudyal AK. Estimation of leisure time physical activity and sedentary behaviour among school adolescents in Nepal. BMC Public Health. 2014;14(1):1449-65.

54. Pelegrini A, Silva DAS, Claumann GS, Cardoso TE, e Silva JMFL, Petroski EL. Practice of walking, moderate and vigorous physical activity and associated factors in adolescents from a state capital of southern Brazil. Revista Brasileira de Cineantropometria e Desempenho Humano. 2014;17(1):11-20.

55. Straatmann VS, Almquist YB, Oliveira AJ, Veiga GV, Rostila M, Lopes CS. Stability and bidirectional relationship between physical activity and sedentary behaviours in Brazilian adolescents: Longitudinal findings from a school cohort study. PLoS One. 2019;14(1).

56. Suyoto PST, Huriyati E, Susilowati R, Julia M. Relative validity of administered indonesian version of the short-form international physical activity questionnaire (IPAQ-SF) among obese adolescent girl population. Pakistan Journal of Nutrition. 2016;15(9):816-20.

57. Wang H, Zhang YF, Xu LL, Jiang CM. Step rate-determined walking intensity and walking recommendation in Chinese young adults: A cross-sectional study. BMJ Open. 2013;3(1).

58. WerneckAndré O, Vancampfort D, Oyeyemi AL, Stubbs B, Silva DR. Associations between TV viewing, sitting time, physical activity and insomnia among 100,839 Brazilian adolescents. Psychiatry Research. 2018;269:700-6.

59. Yildizer G, Yilmaz İ, Novak D. Social capital and physical activity participation among Turkish adolescents in urban centres: A preliminary study. South African Journal for Research in Sport, Physical Education and Recreation. 2019;41(2):117-29.

60. Zhu Z, Tang Y, Zhuang J, Liu Y, Wu X, Cai Y, et al. Physical activity, screen viewing time, and overweight/obesity among Chinese children and adolescents: An update from the 2017 physical activity and fitness in China - The youth study. BMC Public Health. 2019;19(1).

61. Karaca A, Caglar E, Cinemre SA. Physical activity levels of the young adults in an economically developing country: The Turkish sample. Journal of Human Kinetics. 2009;22(1):91-8.

62. Khan A, Burton NW, Trost SG. Patterns and correlates of physical activity in adolescents in Dhaka city, Bangladesh. Public Health. 2017;145:75-82.

63. Silva RC, Malina RM. Level of physical activity in adolescents from Niteroi, Rio de Janeiro, Brazil. Cadernos de Saúde Pública. 2000;16(4):1091-7.

64. Hajian-Tilaki K, Heidari B. Prevalences of overweight and obesity and their association with physical activity pattern among Iranian adolescents aged 12–17 years. Public health nutrition. 2012;15(12):2246-52.

65. Teo PS, Nurul-Fadhilah A, Foo LH. Development of a new computer-based physical activity questionnaire to estimate habitual physical activity level in Malaysian adolescents. Journal of science and medicine in sport. 2013;16(4):327-31.

66. Yelizarova O, Stankevych T, Parats A, Antomonov M, Polka N, Hozak S. Specific Features of the Ukrainian Urban Adolescents' Physical Activity: A Cross-Sectional Study. Journal of Environmental and Public Health. 2020;2020.

67. Ziaei R, Mohammadi R, Dastgiri S, Baybordi E, Rahimi VA, Sadeghi-Bazargani H, et al. The prevalence and correlates of physical activity/inactivity and sedentary behaviour among high-school adolescents in Iran: a cross-sectional study. Journal of Public Health-Heidelberg.

68. Andarge E, Trevethan R, Fikadu T. Assessing the Physical Activity Questionnaire for Adolescents (PAQ-A): Specific and General Insights from an Ethiopian Context. BioMed Research International. 2021;2021.

69. Mohammed OY, Tesfahun E, Ahmed AM, Bayleyegn AD. Self-reported physical activity status among adolescents in Debre Birhan town, Ethiopia: Cross-sectional study. PloS one. 2020;15(2):e0229522.

70. Peralta M, Henriques-Neto D, Bordado J, Loureiro N, Diz S, Marques A. Active Commuting to School and Physical Activity Levels among 11 to 16 Year-Old Adolescents from 63 Low- and Middle-Income Countries. International Journal of Environmental Research and Public Health. 2020;17(4).

71. Nikolić M, Jovanović R, Stanković A. Characteristics of physical activity among healthy serbian adolescents. Revista Brasileira de Medicina do Esporte. 2020;26(1):30-3.

72. Peltzer K. Leisure time physical activity and sedentary behavior and substance use among in-school adolescents in eight African countries. International Journal of Behavioral Medicine. 2010;17(4):271-8.

73. Abu-Mweis SS, Tayyem RF, Bawadi HA, Musaiger AO, Al-Hazzaa HM. Eating habits, physical activity, and sedentary behaviors of Jordanian adolescents' residents of Amman. Mediterranean Journal of Nutrition and Metabolism. 2014;7(1):67-74.

74. El Achhab Y, Marfa A, Echarbaoui I, Chater R, El-Haidani A, Filali-Zegzouti Y. Physical inactivity, sedentary behaviors and dietary habits among Moroccan adolescents in secondary school. Science & Sports. 2018;33(1):58-62.

75. Hamrani A, Mehdad S, El Kari K, El Hamdouchi A, El Menchawy I, Belghiti H, et al. Physical activity and dietary habits among Moroccan adolescents. Public Health Nutrition. 2015;18(10):1793-800.

76. Nascimento-Ferreira MV, De Moraes ACF, Toazza-Oliveira PV, Forjaz CLM, Aristizabal JC, Santaliesra-Pasías AM, et al. Reliability and Validity of a Questionnaire for Physical Activity Assessment in South American Children and Adolescents: The SAYCARE Study. Obesity. 2018;26:S23-S30.

77. Zieff SG, Guedes CM, Wiley J. Youth knowledge of physical activity health benefits: A Brazilian case study. Thescientificworldjournal. 2006;6:1713-21.

78. Amornsriwatanakul A, Lester L, Bull FC, Rosenberg M. Are Thai children and youth sufficiently active? prevalence and correlates of physical activity from a nationally representative cross-sectional study. international journal of behavioral nutrition and physical activity. 2017;14(1):72.

79. Christoph MJ, Grigsby-Toussaint DS, Baingana R, Ntambi JM. Physical Activity, Sleep, and BMI Percentile in Rural and Urban Ugandan Youth. Annals of Global Health. 2017;83(2):311-9.

80. de Lima TR, Silva DAS. Prevalence of physical activity among adolescents in southern Brazil. Journal of Bodywork and Movement Therapies. 2018;22(1):57-63.

81. Florindo AA, Romero A, Peres SV, da Silva MV, Slater B. Development and validation of a physical activity assessment questionnaire for adolescents. Rev Saude Publica. 2006;40(5):802-9.

82. Ben Gharbia H, Gartner A, Traissac P, Delpeuch F, Maire B, El Ati J. A frequency questionnaire to estimate free-living physical activity among Tunisian preadolescent and adolescent children. Public Health Nutrition. 2014;17(10):2253-62.

83. Ghavamzadeh S, Khalkhali HR, Alizadeh M. TV viewing, independent of physical activity and obesogenic foods, increases overweight and obesity in adolescents. Journal of Health, Population & Nutrition. 2013;31(3):334-42.

84. Hong TK, Trang NHHD, van der Ploeg HP, Hardy LL, Dibley MJ. Validity and reliability of a physical activity questionnaire for Vietnamese adolescents. The International Journal of Behavioral Nutrition and Physical Activity. 2012;9.

85. Kee CC, Lim KH, Sumarni MG, Ismail MN, Poh BK, Amal NM. Physical activity and sedentary behaviour among adolescents in petaling district, selangor, malaysia. Malaysian Journal of Medicine and Health Sciences. 2011;7(1):83-93.

86. Micklesfield LK, Pedro TM, Kahn K, Kinsman J, Pettifor JM, Tollman S, et al. Physical activity and sedentary behavior among adolescents in rural South Africa: levels, patterns and correlates. BMC Public Health. 2014;14(1):1-19.

87. Nguyen Hoang Hanh Doan T, Tang Kim H, Van Der Ploeg HP, Hardy LL, Kelly PJ, Dibley MJ. Longitudinal Physical Activity Changes in Adolescents: Ho Chi Minh City Youth Cohort. Medicine & Science in Sports & Exercise. 2012;44(8):1481-9.

88. Prista A, Marques AT, Maia J. Relationship between Physical Activity, Socioeconomic Status, and Physical Fitness of 8-15-Year-Old Youth from Mozambique. American Journal of Human Biology. 1997;9(4):449-57.

89. Silva DAS, Chaput JP, Tremblay MS. Participation frequency in physical education classes and physical activity and sitting time in Brazilian adolescents. PLoS One. 2019;14(3).

90. Su TT, Sim PY, Nahar AM, Majid HA, Murray LJ, Cantwell MM, et al. Association between self-reported physical activity and indicators of body composition in Malaysian adolescents. Prev Med. 2014;67:100-5.

91. Teo PS, Nurul-Fadhilah A, Aziz ME, Hills AP, Foo LH. Lifestyle practices and obesity in Malaysian adolescents. International journal of environmental research and public health. 2014;11(6):5828-38.

92. Verstraeten R, Lachat C, Ochoa-Avilés A, Hagströmer M, Huybregts L, Andrade S, et al. Predictors of validity and reliability of a physical activity record in adolescents. BMC Public Health. 2013;13(1):1-20.

93. Afrifa-Anane E, Agyemang C, Codjoe SNA, Ogedegbe G, Aikins AD. The association of physical activity, body mass index and the blood pressure levels among urban poor youth in Accra, Ghana. BMC Public Health. 2015;15.

94. Barbosa N, Sanchez CE, Patiño E, Lozano B, Thalabard JC, Lebozec S, et al. Quantification of physical activity using the QAPACE questionnaire: A two stage cluster sample design survey of children and adolescents attending urban school. Journal of Sports Medicine and Physical Fitness. 2016;56(5):587-96.

95. Delshad M, Ghanbarian A, Ghaleh NR, Amirshekari G, Askari S, Azizi F. Reliability and validity of the modifiable activity questionnaire for an Iranian urban adolescent population. International Journal of Preventive Medicine. 2015;2015-January.

96. TudorLocke C, Ainsworth BE, Adair LS, Du S, Popkin BM. Physical activity and inactivity in Chinese school-aged youth: the China Health and Nutrition Survey. International Journal of Obesity & Related Metabolic Disorders. 2003;27(9):1093.

97. Bastos JP. Prevalence of sedentary lifestyle and associated factors in Brazilian adolescents. Pelotas: Universidade Federal de Pelotas; 2006.

98. Bastos JP, Araújo CLP, Hallal PC. Prevalence of insufficient physical activity and associated factors in Brazilian adolescents. Journal of physical activity and health. 2008;5(6):777-94.

99. Micklesfield LK, Pedro TM, Kahn K, Kinsman J, Pettifor JM, Tollman S, et al. Physical activity and sedentary behavior among adolescents in rural South Africa: levels, patterns and correlates. BMC public health. 2014;14(1):1-10.

100. Mehreen TS, Ranjani H, Anitha C, Jagannathan N, Pratt M, Mohan V, et al. Reliability and Validity of a Physical Activity Questionnaire for Indian Children and Adolescents. Indian Pediatrics. 2020;57(8):707-11.

101. Ferrari G, Rezende LF, Wagner GA, Florindo AA, Peres MFT. Physical activity patterns in a representative sample of adolescents from the largest city in Latin America: a cross-sectional study in Sao Paulo. BMJ open. 2020;10(9):e037290.

102. Coll CDVN, Knuth AG, Bastos JP, Hallal PC, Bertoldi AD. Time trends of physical activity among Brazilian adolescents over a 7-year period. Journal of Adolescent Health. 2014;54(2):209-13.

103. Alberico CO, Schipperijn J, Reis RS. Use of global positioning system for physical activity research in youth: ESPAÇOS Adolescentes, Brazil. Prev Med. 2017;103:S59-S65.

104. Bénéfice E, Cames C. Physical activity patterns of rural Senegalese adolescent girls during the dry and rainy seasons measured by movement registration and direct observation methods. European journal of clinical nutrition. 1999;53(8):636-43.

105. Benefice E, Garnier D, Ndiaye G. Assessment of physical activity among rural Senegalese adolescent girls: Influence of age, sexual maturation, and body composition. Journal of Adolescent Health. 2001;28(4):319-27.

106. Cook I. Do low levels of physical activity in female adolescents cause overweight and obesity? Objectively measured physical activity levels of periurban and rural adolescents. South African Medical Journal. 2015;105(8):659-63.

107. Corder K, Brage S, Ramachandran A, Snehalatha C, Wareham N, Ekelund U. Comparison of two Actigraph models for assessing free-living physical activity in Indian adolescents. Journal of Sports Sciences. 2007;25(14):1607-11.

108. da Costa BGG, da Silva KS, Malheiros LEA, Minatto G, de Lima LRA, Petroski EL. Are adolescents really being sedentary or inactive when at school? An analysis of sedentary behaviour and physical activity bouts. European Journal of Pediatrics. 2018;177(11):1705-10.

109. Konharn K, Eungpinichpong W, Promdee K, Sangpara P, Nongharnpitak S, Malila W, et al. Validity and reliability of smartphone applications for the assessment of walking and running in normal-weight and overweight/obese young adults. Journal of Physical Activity and Health. 2016;13(12):1333-40.

110. Nie MJ, Fan CQ, Sun RZ, Wang JJ, Feng Q, Zhang YF, et al. Accelerometer-measured physical activity in children and adolescents at altitudes over 3500 meters: A cross-sectional study in tibet. International Journal of Environmental Research and Public Health. 2019;16(5).

111. Ojiambo RM, Easton C, Casajús JA, Konstabel K, Reilly JJ, Pitsiladis Y. Effect of urbanization on objectively measured physical activity levels, sedentary time, and indices of adiposity in Kenyan adolescents. Journal of Physical Activity and Health. 2012;9(1):115-23.

112. Sayre MK, Pike IL, Raichlen DA. High levels of objectively measured physical activity across adolescence and adulthood among the Pokot pastoralists of Kenya. American Journal of Human Biology. 2019;31(1).

113. Naseer S. Pedometer determined Physical Activity levels and Reliability of Pedometer data in Pakistani Adolescents. Pakistan Journal of Public Health. 2020;10(3):190-6.

114. Wushe SN, Moss SJ, Monyeki MA. Objectively determined habitual physical activity in South African adolescents: the PAHL study. BMC Public Health. 2014;14(1):284-99.

115. Corder K, Brage S, Wright A, Ramachandran A, Snehalatha C, Yamuna A, et al. Physical activity energy expenditure of adolescents in India. Obesity. 2010;18(11):2212-9.

116. da Silva ICM, Hino AA, Lopes A, Ekelund U, Brage S, Goncalves H, et al. Built environment and physical activity: domain- and activity-specific associations among Brazilian adolescents. BMC Public Health. 2017;17.

117. Prista A, Nhantumbo L, Saranga S, Lopes V, Maia J, Seabra A, et al. Physical activity assessed by accelerometry in rural african school-age children and adolescents. Pediatr Exerc Sci. 2009;21(4):384-99.

118. Hallal PC, Reichert FF, Clark VL, Cordeira KL, Menezes AMB, Eaton S, et al. Energy Expenditure Compared to Physical Activity Measured by Accelerometry and Self-Report in Adolescents: A Validation Study. PLoS One. 2013;8(11):1-7.

119. Prista A, Marques AT, Maia JAR. Empirical validation of an instrument to measure habitual physical activity in youth from Maputo, Mozambique. American Journal of Human Biology. 2000;12(4):437-46.

120. Tudor-Locke C, Ainsworth BE, Adair LS, Popkin BM. Physical activity in Filipino youth: The Cebu Longitudinal Health and Nutrition Survey. International Journal of Obesity. 2003;27(2):181-90.

121. Aniza I, Fairuz M. Factors influencing physical activity level among secondary school adolescents in Petaling District, Selangor. The Medical Journal of Malaysia. 2009;64(3):228-32.

122. Gouthon P, Falola J, Aremou M, Dagba J, Tossou J, Legba J, et al. Comparison of physical activity among Beninese adolescents attending schools in rural, suburban and urban areas: physical education and health. African Journal for Physical Health Education, Recreation and Dance. 2007;13(2):196-208.

123. Hovsepian S, Kelishadi R, Motlagh ME, Kasaeian A, Shafiee G, Arefirad T, et al. Level of physical activity and screen time among Iranian children and adolescents at the national and provincial level: The CASPIAN-IV study. Medical journal of the Islamic Republic of Iran. 2016;30:422.

124. Akman M, Akan H, Izbirak G, Tanriover O, Tilev SM, Yildiz A, et al. Eating patterns of Turkish adolescents: a cross-sectional survey. Nutr J. 2010;9.

125. Araki EL, Philippi ST, Martinez MF, Estima CCP, Leal GVS, Alvarenga MS. Pattern of meals eaten by adolescents from technical schools of São Paulo, SP, Brazil. Rev Paul Pediatr. 2011;29(2):164-70.

126. Can HO, Ceber E, Sogukpinar N, Saydam BK, Otles S, Ozenturk G. Eating habits, knowledge about cancer prevention and the HPLP scale in Turkish adolescents. Asian Pacific Journal of Cancer Prevention. 2008;9(4):569-74.

127. Ghasab Shirazi M, Kazemi A, Kelishadi R, Mostafavi F. The Improvement of Dietary Behaviors among Iranian Adolescent Girls: A Theory-Based Randomized Controlled Trial. Health Educ Res. 2019;34(2):159-72.

128. Amos PM, Intiful FD, Boateng L. Factors that were found to influence Ghanaian Adolescents' Eating Habits. Sage Open. 2012;2(4):1-6.

129. Gitau TM, Micklesfield LK, Pettifor JM, Norris SA. Changes in Eating Attitudes, Body Esteem and Weight Control Behaviours during Adolescence in a South African Cohort. PLoS ONE. 2014;9(10):1-10.

130. Maulida R, Nanishi K, Green J, Shibanuma A, Jimba M. Food-choice motives of adolescents in Jakarta, Indonesia: the roles of gender and family income. Public Health Nutrition. 2016;19(15):2760-8.

131. Sahingoz SA, Sanlier N. Compliance with Mediterranean Diet Quality Index (KIDMED) and nutrition knowledge levels in adolescents. A case study from Turkey. Appetite. 2011;57(1):272-7.

132. Steff M, Verney J, Marinau M, Perte S, Pereira B, Bryant E, et al. Toward a Romanian version of the Three-Factor Eating Questionnaire-R21 for children and adolescents (CTFEQ-R21): Preliminary psychometric analysis and relation with body composition. Developmental period medicine. 2019;23(1):45-53.

133. Loh DA, Moy FM, Zaharan NL, Mohamed Z. Eating Behaviour among Multi-Ethnic Adolescents in a Middle-Income Country as Measured by the Self-Reported Children’s Eating Behaviour Questionnaire. PLoS ONE. 2013;8(12):1-.

134. Ndagire CT, Muyonga JH, Nakimbugwe D. Fruit and vegetable consumption, leisure-time physical activity, and sedentary behavior among children and adolescent students in Uganda. Food Science and Nutrition. 2019;7(2):599-607.

135. Zahedi H, Kelishadi R, Heshmat R, Motlagh ME, Ranjbar SH, Ardalan G, et al. Association between junk food consumption and mental health in a national sample of Iranian children and adolescents: The CASPIAN-IV study. Nutrition. 2014;30(11/12):1391-7.

136. Marchioni DML, Voci SM, De Lima FEL, Fisberg RM, Slater B. Reproducibility of a food frequency questionnaire for adolescents. Cadernos de Saude Publica. 2007;23(9):2187-96.

137. Ogunsile SE, Ogundele BO. Effect of game-enhanced nutrition education on knowledge, attitude and practice of healthy eating among adolescents in Ibadan, Nigeria. International Journal of Health Promotion & Education. 2016;54(5):207-16.

138. Morales A, Montilva M, Gómez N, Cordero M. Adaptación transcultural de la escala de evaluación de conductas alimentarias en adolescentes: "Adolescent food habits checklist". Trans-cultural adaptation of the eating behaviors rating scale in adolescents: "Adolescent food habits checklist". 2012;25(1):25-33.

139. Whati LH. The development of a valid and reliable nutrition knowledge questionnaire and performance-rating scale for urban South African adolescents participating in the 'birth-to-twenty' study. 2005.

140. Vakili M, Abedi P, Sharifi M, Hosseini M. Dietary diversity and its related factors among adolescents: a survey in Ahvaz-Iran. Global journal of health science. 2013;5(2):181-6.

141. Neutzling MB, Assuncao MCF, Malcon MC, Hallal PC, Menezes AMB. Food habits of adolescent students from Pelotas, Brazil. Revista De Nutricao-Brazilian Journal of Nutrition. 2010;23(3):379-88.

142. Correa RD, Vencato PH, Rockett FC, Bosa VL. Dietary patterns: are there differences between children and adolescents? Ciencia & Saude Coletiva. 2017;22(2):553-62.

143. Melaku Y, Dirar A, Feyissa GT, Tamiru D. Optimal dietary practices and nutritional knowledge of school adolescent girls in Jimma Town, South West Ethiopia. International Journal of Adolescence & Youth. 2018;23(3):299-307.

144. Li M, Dibley MJ, Sibbritt DW, Yan H. Dietary habits and overweight/obesity in adolescents in Xi'an City, China. 2010. p. 76-82.

145. Majid HA, Ramli L, Ying SP, Su TT, Jalaludin MY, Mohsein N. Dietary Intake among Adolescents in a Middle-Income Country: An Outcome from the Malaysian Health and Adolescents Longitudinal Research Team Study (the MyHeARTs Study). PLoS One. 2016;11(5).

146. Monika Arora MA, Nazar GP, Gupta VK, Perry CL, Reddy KS, Stigler MH. Association of breakfast intake with obesity, dietary and physical activity behavior among urban school-aged adolescents in Delhi, India: results of a cross-sectional study. BMC Public Health. 2012;12(1):881-92.

147. Olumakaiye MF, Atinmo T, Olubayo-Fatiregun MA. Food Consumption Patterns of Nigerian Adolescents and Effect on Body Weight. Journal of Nutrition Education & Behavior. 2010;42(3):144-51.

148. de Assumpção D, Barros MBA, Fisberg RM, Carandina L, Goldbaum M, Cesar CLG. Diet quality among adolescents: A population-based study in Campinas, Brazil. Revista Brasileira de Epidemiologia. 2012;15(3):605-16.

149. Jikamo B, Samuel M. Does dietary diversity predict the nutritional status of adolescents in Jimma Zone, Southwest Ethiopia? BMC Research Notes. 2019;12(1):N.PAG-N.PAG.

150. Shaikh NI, Frediani JK, Ramakrishnan U, Patil SS, Yount KM, Martorell R, et al. Development and evaluation of a Nutrition Transition-FFQ for adolescents in South India. Public Health Nutr. 2017;20(7):1162-72.

151. Ali Z, Abizari A-R. Ramadan fasting alters food patterns, dietary diversity and body weight among Ghanaian adolescents. Nutrition journal. 2018;17(1):1-14.

152. Choeda T, Jeyashree K, Kathirvel S, Dorji T, Dorjee K, Tenzin K, et al. Dietary behavior of school-going adolescents in Bhutan: Findings from the global school-based student health survey in 2016. Nutrition. 2021;90.

153. Li L, Sun N, Zhang L, Xu G, Liu J, Hu J, et al. Fast food consumption among young adolescents aged 12–15 years in 54 low- and middle-income countries. Global Health Action. 2020;13(1).

154. Pajuelo SR, Saintila J, Vásquez MR, Calizaya-Milla YE. Knowledge, attitudes and practices about healthy eating in a Peruvian adolescent population: A cross-sectional study. Revista Espanola de Nutricion Comunitaria. 2021;27(2).

155. Slater B, Enes CC, López RVM, Damasceno NRT, Voci SM. Validation of a food frequency questionnaire to assess the consumption of carotenoids, fruits and vegetables among adolescents: The method of triads. Cadernos de Saude Publica. 2010;26(11):2090-100.

156. Dalky HF, Al Momani MH, Al-Drabaah TK, Jarrah S. Eating Habits and Associated Factors Among Adolescent Students in Jordan. Clinical Nursing Research. 2017;26(4):538-52.

157. Ochoa-Avilés A, Verstraeten R, Lachat C, Andrade S, Van Camp J, Donoso S, et al. Dietary intake practices associated with cardiovascular risk in urban and rural Ecuadorian adolescents: a cross-sectional study. BMC Public Health. 2014;14(1):939-.

158. AllehdanSabika S, Tayyem RF, Bawadi HA, Al-Awwad NJ, Al-Mannai M, Musaiger AO. Fast foods perception among adolescents by gender and weight status. Nutrition and Health. 2017;23(1):39-45.

159. Aounallah-Skhiri H, Traissac P, Ati JE, Eymard-Duvernay S, Landais E, Achour N, et al. Nutrition transition among adolescents of a south-Mediterranean country: dietary patterns, association with socio-economic factors, overweight and blood pressure. A cross-sectional study in Tunisia. Nutrition Journal. 2011;10:38-54.

160. Azadbakht L, Akbari F, Esmaillzadeh A. Diet quality among Iranian adolescents needs improvement. Public Health Nutrition. 2015;18(4):615-21.

161. Azeredo CM, de Rezende LFM, Canella DS, Claro RM, Peres MFT, Luiz OD, et al. Food environments in schools and in the immediate vicinity are associated with unhealthy food consumption among Brazilian adolescents. Prev Med. 2016;88:73-9.

162. Borges CA, Enes CC, Slater B, Conde WL. BMI Changes Associated With Dietary Trends Among Brazilian Adolescents. Infant, Child, and Adolescent Nutrition. 2012;4(6):361-8.

163. Ahadi Z, Qorbani M, Kelishadi R, Ardalan G, Motlagh ME, Asayesh H, et al. Association between breakfast intake with anthropometric measurements, blood pressure and food consumption behaviors among Iranian children and adolescents: the CASPIAN-IV study. Public Health (Elsevier). 2015;129(6):740-7.

164. Al Sabbah H, Vereecken C, Kolsteren P, Abdeen Z, Maes L. Food habits and physical activity patterns among Palestinian adolescents: Findings from the national study of Palestinian schoolchildren (HBSC-WBG2004). Public Health Nutr. 2007;10(7):739-46.

165. Alavi M, Eftekhari MB, Noot R, Rafinejad J, Chinekesh A. Dietary habits among adolescent girls and their association with parental educational levels. Global journal of health science. 2013;5(5):202-6.

166. Barufaldi LA, Abreu GD, Oliveira JS, dos Santos DF, Fujimori E, Vasconcelos SML, et al. ERICA: prevalence of healthy eating habits among Brazilian adolescents. Rev Saude Publica. 2016;50.

167. Barufaldi LA, Abreu GDA, Veiga GVD, Sichieri R, Kuschnir MCC, Cunha DB, et al. Software to record 24-hour food recall: Application in the Study of Cardiovascular Risks in Adolescents. Revista Brasileira de Epidemiologia. 2016;19(2):464-8.

168. Belachew T, Lindstrom D, Gebremariam A, Hogan D, Lachat C, Huybregts L, et al. Food Insecurity, Food Based Coping Strategies and Suboptimal Dietary Practices of Adolescents in Jimma Zone Southwest Ethiopia. PLoS ONE. 2013;8(3):1-9.

169. Cacavas K, Mavoa H, Kremer P, Malakellis M, Fotu K, Swinburn B, et al. Tongan adolescents' eating patterns: opportunities for intervention. 2011. p. 24-33.

170. Cai-Xia Z, Yu-Ming C, Wei-Qing C, Yi-Xiang S, Cui-Ling W, Jiang-Nan W. Food group intake among adolescents in Guangzhou city compared with the Chinese dietary guidelines. Asia Pacific Journal of Clinical Nutrition. 2012;21(3):450-6.

171. Itani L, Chatila H, Dimassi H, El Sahn F. Development and validation of an Arabic questionnaire to assess psychosocial determinants of eating behavior among adolescents: a cross-sectional study. J Health Popul Nutr. 2017;36(1):10.

172. Joulaei H, Keshani P, Kaveh MH. Nutrition literacy as a determinant for diet quality amongst young adolescents: a cross sectional study. Progress in Nutrition. 2018;20(3):455-64.

173. Feeley AB, Norris SA. Added sugar and dietary sodium intake from purchased fast food, confectionery, sweetened beverages and snacks among Sowetan adolescents. SAJCH South African Journal of Child Health. 2014;8(3):88-91.

174. Feeley A, Musenge E, Pettifor JM, Norris SA. Changes in dietary habits and eating practices in adolescents living in urban South Africa: The birth to twenty cohort. Nutrition. 2012;28(7/8):e1-e6.

175. Francis DK, Van den Broeck J, Younger N, McFarlane S, Rudder K, Gordon-Strachan G, et al. Fast-food and sweetened beverage consumption: association with overweight and high waist circumference in adolescents. Public Health Nutrition. 2009;12(8):1106-14.

176. Garba JA, Rampal L, Hejar AR, Salmiah MS. Major dietary patterns and their associations with socio-demographic characteristics and obesity among adolescents in Petaling District, Malaysia. Malaysian Journal of Medicine and Health Sciences. 2014;10(1):13-21.

177. Korkalo L, Erkkola M, Heinonen AE, Freese R, Selvester K, Mutanen M. Associations of dietary diversity scores and micronutrient status in adolescent Mozambican girls. European Journal of Nutrition. 2017;56(3):1179-89.

178. Macuácua ME, Taconeli CA, Osório MM. Dietary patterns and associated socio-economic factors in rural mozambican adolescents. Malawi Medical Journal. 2019;31(1):25-30.

179. Mikki N, Abdul-Rahim HF, Shi Z, Holmboe-Ottesen G. Dietary habits of Palestinian adolescents and associated sociodemographic characteristics in Ramallah, Nablus and Hebron governorates. Public Health Nutr. 2010;13(9):1419-29.

180. Mirmiran P, Azadbakht L, Azizi F. Dietary behaviour of Tehranian adolescents does not accord with their nutritional knowledge. Public Health Nutrition. 2007;10(9):897-901.

181. Mohamed K, Tin Su T, Muhammad Yazid J, Al-Sadat N, Hazreen Abdul M, Tin TS, et al. Comparative validity of a Food Frequency Questionnaire (MyUM Adolescent FFQ) to estimate the habitual dietary intake of adolescents in Malaysia. Asia Pacific Journal of Clinical Nutrition. 2018;27(4):898-907.

182. Musaiger AO, Kalam F. Dietary habits and lifestyle among adolescents in Damascus, Syria. Annals of Agricultural and Environmental Medicine. 2014;21(2):416-9.

183. Ortiz-Hernández L, Gómez-Tello BL. Food consumption in Mexican adolescents. Consumo de alimentos en adolescentes mexicanos. 2008;24(2):127-35.

184. Patcheep K. FACTORS INFLUENCING URBAN ADOLESCENTS' EATING BEHAVIOR, RATCHABURI PROVINCE, THAILAND: AN APPLICATION OF THE THEORY OF PLANNED BEHAVIOR. Journal of Health Research. 2015;29(6):441-7.

185. Raghunatha Rao D, Vijayapushpam T, Subba Rao GM, Antony GM, Sarma KVR. Dietary habits and effect of two different educational tools on nutrition knowledge of school going adolescent girls in Hyderabad, India. European Journal of Clinical Nutrition. 2007;61(9):1081-5.

186. Ranjana S, Mahomoodally FM, Ramasawmy D. Is healthy eating behaviour common among school adolescents in mauritius? Current Research in Nutrition and Food Science. 2013;1(1):11-22.

187. Rodriguez CA, Smith ER, Villamor E, Zavaleta N, Respicio-Torres G, Contreras C, et al. Development and validation of a food frequency questionnaire to estimate intake among children and adolescents in Urban Peru. Nutrients. 2017;9(10).

188. Roba AC, Gabriel-Micheal K, Zello GA, Jaffe J, Whiting SJ, Henry CJ. A Low Pulse Food Intake May Contribute to the Poor Nutritional Status and Low Dietary Intakes of Adolescent Girls in Rural Southern Ethiopia. Ecology of Food & Nutrition. 2015;54(3):240-54.

189. Saravia L, González-Zapata LI, Rendo-Urteaga T, Ramos J, Collese TS, Bove I, et al. Development of a Food Frequency Questionnaire for Assessing Dietary Intake in Children and Adolescents in South America. Obesity. 2018;26:S31-S40.

190. SedibeModiehi H, Pisa PT, Feeley AB, Pedro TM, Kahn K, Norris SA. Dietary Habits and Eating Practices and Their Association with Overweight and Obesity in Rural and Urban Black South African Adolescents. Nutrients. 2018;10(2).

191. Seubsman SA, Kelly M, Yuthapornpinit P, Sleigh A. Cultural resistance to fast-food consumption? A study of youth in North Eastern Thailand. International Journal of Consumer Studies. 2009;33(6):669-75.

192. Schneider BC, Dumith SdC, Lopes C, Severo M, Assunção MCF. How Do Tracking and Changes in Dietary Pattern during Adolescence Relate to the Amount of Body Fat in Early Adulthood? PLoS ONE. 2016;11(2):1-11.

193. Soyer MT, Ergin I, Gursoy ST. Effects of social determinants on food choice and skipping meals among Turkish adolescents. Asia Pacific Journal of Clinical Nutrition. 2008;17(2):208-15.

194. Venter I, Winterbach A. Dietary fat knowledge and intake of mid-adolescents attending public schools in the Bellville/Durbanville area of the Western Cape Province. South African Journal of Clinical Nutrition. 2014;23(2).

195. Wang L, Zhang Z, Chang Y, Wang X, Hou M, Wei J, et al. Comparison of dietary habits between migrant and local adolescents in Shenzhen, China. Asia Pacific Journal of Clinical Nutrition. 2011;20(4):624-31.

196. Williams J, Townsend N, Rayner M, Jayawardena R, Katulanda P, Manoharan S, et al. Diet quality of adolescents in rural Sri Lanka based on the Diet Quality Index–International: findings from the 'Integrating Nutrition Promotion and Rural Development' project. Public Health Nutrition. 2019;22(10):1735-44.

197. Araujo MC, Yokoo EM, Pereira RA. Validation and Calibration of a Semiquantitative Food Frequency Questionnaire Designed for Adolescents. Journal of the American Dietetic Association. 2010;110(8):1170-7.

198. Abudayya AH, Stigum H, Shi Z, Abed Y, Holmboe-Ottesen G. Sociodemographic correlates of food habits among school adolescents (12-15 year) in north Gaza Strip. BMC Public Health. 2009;9.

199. Areekul W, Viravathana N, Aimpun P, Watthanakijthavongkul K, Khruacharooen J, Awaiwanont A, et al. Dietary behaviors and nutritional status of adolescents in a remote rural area of Thailand. Journal of the Medical Association of Thailand = Chotmaihet thangphaet. 2005;88 Suppl 3:S240-6.

200. Chiarelli G, Ulbrich AZ, Bertin RL. Body composition and dietary intake of adolescents from public schools in Blumenau (Brazil). Revista Brasileira de Cineantropometria e Desempenho Humano. 2011;13(4):265-71.

201. Henn RL, Fuchs SC, Moreira LB, Fuchs FD. Development and validation of a food frequency questionnaire (FFQ-Porto alegre) for adolescent, adult and elderly populations from Southern Brazil. Cadernos de Saude Publica. 2010;26(11):2068-79.

202. Niu J, Seo D-C, Lohrmann D. Weight Perception and Dietary Intake among Chinese Youth, 2004-2009. International Journal of Behavioral Medicine. 2014;21(4):691-9.

203. Rodríguez LA, Mundo‐Rosas V, Méndez‐Gómez‐Humarán I, Pérez‐Escamilla R, Shamah‐Levy T. Dietary quality and household food insecurity among Mexican children and adolescents. Maternal & Child Nutrition. 2017;13(4):n/a-N.PAG.

204. Singh JK, Acharya D, Gautam S, Adhikari M, Park JH, Yoo SJ, et al. Socio-Demographic and Diet-Related Factors Associated with Insufficient Fruit and Vegetable Consumption among Adolescent Girls in Rural Communities of Southern Nepal. International Journal of Environmental Research and Public Health. 2019;16(12).

205. Wate JT, Snowdon W, Millar L, Nichols M, Mavoa H, Goundar R, et al. Adolescent dietary patterns in Fiji and their relationships with standardized body mass index. International Journal of Behavioral Nutrition & Physical Activity. 2013;10:45-56.

206. Abizari AR, Ali Z. Dietary patterns and associated factors of schooling Ghanaian adolescents. J Health Popul Nutr. 2019;38(1):5.

207. Bakir BO, Akan H, Akman M, Zahmacioglu O, Hayran O. Nutritional status, healthy eating index and eating attitudes of the adolescents in Istanbul: A cross-sectional study. Int J Adolesc Med Health. 2015;2015.

208. Bardosono S, Monrozier R, Permadhi I, Manikam NRM, Pohan R, Guelinckx I. Total fluid intake assessed with a 7-day fluid record versus a 24-h dietary recall: a crossover study in Indonesian adolescents and adults. European Journal of Nutrition. 2015;54:17-25.

209. Birru SM, Tariku A, Belew AK. Improved dietary diversity of school adolescent girls in the context of urban Northwest Ethiopia: 2017. Italian Journal of Pediatrics. 2018;44(1):N.PAG-N.PAG.

210. Borges CA, Marchioni DML, Levy RB, Slater B. Dietary patterns associated with overweight among Brazilian adolescents. Appetite. 2018;123:402-9.

211. Bullecer ER, Rabuco LB, Aninao DAB, De Roxas RC, Esguerra JCA, Lim PRU, et al. Dietary diversity score as an indicator of nutritional adequacy of diets among 16-19-year-old adolescents. Acta Medica Philippina. 2012;46(1):28-33.

212. Cunha DB, Bezerra IN, Pereira RA, Sichieri R. At-home and away-from-home dietary patterns and BMI z-scores in Brazilian adolescents. Appetite. 2018;120:374-80.

213. da Costa Louzada ML, Baraldi LG, Steele EM, Bortoletto Martins AP, Canella DS, Moubarac J-C, et al. Consumption of ultra-processed foods and obesity in Brazilian adolescents and adults. Preventive Medicine: An International Journal Devoted to Practice and Theory. 2015;81:9-15.

214. Das G, Pathania R, Das G. Assessment of dietary pattern of adolescents in Himachal Pradesh of India. Journal of Paramedical Sciences. 2016;7(3):1-7.

215. de Andrade SC, Barros MBD, Carandina L, Goldbaum M, Cesar CLG, Fisberg RM. Dietary Quality Index and Associated Factors among Adolescents of the State of Sao Paulo, Brazil. Journal of Pediatrics. 2010;156(3):456-60.

216. de Morais CMM, Pinheiro LGB, Lima S, Lyra CO, Evangelista K, Lima KC, et al. Dietary patterns of young adolescents in urban areas of Northeast Brazil. Nutricion Hospitalaria. 2013;28(6):1977-84.

217. Dixit S, Singh JV, Kant S, Agarwal GG, Dubey A, Kumari N. A cross-sectional study on predictors and significance of eating behavior of adolescent girls. Vulnerable Children & Youth Studies. 2014;9(1):10-6.

218. Esmaillzadeh A, Alizadeh M, Mohtadinia J, Pourghasem-Gargari B. Major dietary patterns among female adolescent girls of Talaat intelligent guidance school, Tabriz, Iran. Iran Red Crescent MedJ. 2012;14(7):436-41.

219. Estrada-Reyes C, Tlatempa-Sotelo P, Valdés-Ramos R, Cabañas-Armesilla M, Manjarrez-Montes-De-Oca R. Dietary Patterns and Fitness Level in Mexican Teenagers. Journal of Nutrition and Metabolism. 2018;2018.

220. Funke OM, Ajayi A, Ajayi OA. Determinants of food choices of adolescents in south-western Nigeria. African Journal of Food, Agriculture, Nutrition and Development. 2007;7(6):6-.

221. Garipagaoglu M, Oner N, Vatansever U, Inan M, Kucukugurluoglu Y, Turan C. Dietary Intakes of Adolescents Living in Edirne, Turkey. Journal of the American College of Nutrition. 2008;27(3):394-400.

222. Korkalo L, Freese R, Fidalgo L, Selvester K, Ismael C, Mutanen M. A cross-sectional study on the diet and nutritional status of adolescent girls in zambézia province, mozambique (the zane study): Design, methods, and population characteristics. J Med Internet Res. 2014;16(3).

223. Mascarenhas JMO, Silva RDCR, Machado MEPC, Santos CADST, Marchioni DML, Barreto ML. Validation of a food frequency questionnaire designed for adolescents in Salvador, Bahia, Brazil. Rev Nutr. 2016;29:163-71.

224. Magbuhat RMT, Borazon EQ, Villarino BJ. Food preferences and dietary intakes of Filipino adolescents in Metro Manila, The Philippines. Malaysian Journal of Nutrition. 2011;17(1):31-41.

225. Majid HA, Amiri M, Mohd Azmi N, Su TT, Jalaludin MY, Al-Sadat N. Physical activity, body composition and lipids changes in adolescents: analysis from the MyHeART Study. Scientific reports. 2016;6(1):1-8.

226. Mascarenhas JMO, Silva RDCR, De Assis AMO, De Santana MLP, De Moraes LTLP, Barreto ML. Identification of food intake patterns and associated factors in teenagers. Rev Nutr. 2014;27(1):45-54.

227. Mirmiran P, Azadbakht L, Esmaillzadeh A, Azizi F. Dietary diversity score in adolescents - A good indicator of the nutritional adequacy of diets: Tehran lipid and glucose study. Asia Pacific Journal of Clinical Nutrition. 2004;13(1):56-60.

228. Nabhani-Zeidan M, Naja F, Nasreddine L. Dietary intake and nutrition-related knowledge in a sample of Lebanese adolescents of contrasting socioeconomic status. Food and Nutrition Bulletin. 2011;32(2):75-83.

229. Monge-Rojas R. Dietary intake as a cardiovascular risk factor in Costa Rican adolescents. Journal of Adolescent Health. 2001;28(4):328-37.

230. Napier CE, Hlambelo N. Contribution of school lunchboxes to the daily food intake of adolescent girls in Durban. SAJCH South African Journal of Child Health. 2014;8(2):59-63.

231. Neto ACB, De Andrade MIS, De Menezes Lima VL, Diniz ADS. Body weight and food consumption scores in adolescents from northeast Brazil. Rev Paul Pediatr. 2015;33(3):319-26.

232. Nithya DJ, Bhavani RV. DIETARY DIVERSITY AND ITS RELATIONSHIP WITH NUTRITIONAL STATUS AMONG ADOLESCENTS AND ADULTS IN RURAL INDIA. Journal of Biosocial Science. 2018;50(3):397-413.

233. Olumakaiye MF, Ajayi A. Determinants of food choices of adolescents in south-western Nigeria. African Journal of Food, Agriculture, Nutrition and Development. 2007;7(6).

234. Ogunkunle MO, Oludele AS. Food intake and meal pattern of adolescents in school in Ila Orangun, south-west Nigeria. South African Journal of Clinical Nutrition. 2013;26(4):188-93.

235. Oldewage-Theron W, Egal A, Moroka T. Nutrition Knowledge and Dietary Intake of Adolescents in Cofimvaba, Eastern Cape, South Africa. Ecol Food Nutr. 2015;54(2):138-56.

236. Onyiriuka AN, Umoru DD, Ibeawuchi AN. Weight status and eating habits of adolescent Nigerian urban secondary school girls. South African Journal of child health. 2013;7(3):108-11.

237. Onyiriuka AN, Ibeawuchi AN, Onyiriuka RC. Assessment of eating habits among adolescent nigerian urban secondary schoolgirls. Sri Lanka Journalof Child Health. 2013;42(1):20-6.

238. Oogarah-Pratap B. Dietary habits of Mauritian school adolescents. Nutrition and Food Science. 2007;37(6):442-51.

239. Otuneye AT, Ahmed PA, Abdulkarim AA, Aluko OO, Shatima DR. Relationship between dietary habits and nutritional status among adolescents in Abuja municipal area council of Nigeria. 2017. p. 128-35.

240. Pangan MRL, Dela Cruz KKL, Nachura MSC, Padolina JL, Ramos MM, Sadorra AQ. Dietary energy density and fast food consumption of 16-21 year-old adolescents. Acta Medica Philippina. 2012;46(3):75-80.

241. Rezali FW, Chin YS, Shariff ZM, Mohd Yusof BN, Sanker K, Woon FC. Evaluation of diet quality and its associated factors among adolescents in Kuala Lumpur, Malaysia. Nutrition Research and Practice. 2015;9(5):511-6.

242. Rouhani MH, Mirseifinezhad M, Omrani N, Esmaillzadeh A, Azadbakht L. Fast food consumption, quality of diet, and obesity among Isfahanian adolescent girls. Journal of Obesity. 2012;2012.

243. Ronca DB, Blume CA, Cureau FV, Camey SA, Leotti VB, Drehmer M, et al. Diet quality index for Brazilian adolescents: the ERICA study. European Journal of Nutrition. 2019.

244. Samaranayaka S, Perera A, Warnasuriya N, Aiyas S. Food habits among adolescents in Colombo, Sri Lanka. Middle East Journal of Family Medicine. 2013;11(6):26-34.

245. Som N, Mishra SK, Mukhopadhyay S. Weight concerns and food habits of adolescent girls in two contrasting ecological regions: A comparative study in India. Eating Behaviors. 2016;20:21-6.

246. Tayel DI, El-Sayed NA, El-Sayed NA. Dietary pattern and blood pressure levels of adolescents in Sohag, Egypt. Journal of the Egyptian Public Health Association. 2013;88(2):97-103.

247. Tehrani AN, Farhadnejad H, Salehpour A, Beyzai B, Hekmatdoost A, Rashidkhani B. The association between nutrition knowledge and adherence to a Mediterranean dietary pattern in Iranian female adolescents. Int J Adolesc Med Health. 2019.

248. Tek NA, Yildiran H, Akbulut G, Bilici S, Koksal E, Karadag MG, et al. Evaluation of dietary quality of adolescents using Healthy Eating Index. Nutrition Research and Practice. 2011;5(4):322-8.

249. Wiecha JM, Hebert JR, Lim M. Diet measurement in VietNamese youth: Concurrent reliability of a self-administered food frequency questionnaire. J Community Health. 1994;19(3):181-8.

250. Wei X, Caihong S, Li Z, Xin Z, Jiajia W, Hui W, et al. Reproducibility and Relative Validity of a Food Frequency Questionnaire Developed for Female Adolescents in Suihua, North China. PLoS ONE. 2011;6(5):1-7.

251. El-Gilany A-H, Elkhawaga G. Socioeconomic determinants of eating pattern of adolescent students in Mansoura, Egypt. Pan African Medical Journal. 2012;13(1).

252. Ahmed F, Zareen M, Khan MR, Banu CP, Haq MN, Jackson AA. Dietary pattern, nutrient intake and growth of adolescent school girls in urban Bangladesh. Public health nutrition. 1998;1(2):83-92.

253. Kalkan I. Dietary habits of Turkish adolescents in Konya, Turkey. New Trends and Issues Proceedings on Advances in Pure and Applied Sciences. 2016(7):190-6.

254. Alam N, Roy SK, Ahmed T, Ahmed AS. Nutritional status, dietary intake, and relevant knowledge of adolescent girls in rural Bangladesh. J Health Popul Nutr. 2010;28(1):86.

255. Alangea DO, Aryeetey RN, Gray HL, Laar AK, Adanu RM. Dietary patterns and associated risk factors among school age children in urban Ghana. BMC nutrition. 2018;4(1):1-9.

256. Amos PM, Intiful FD, Boateng L. Factors that were found to influence Ghanaian adolescents’ eating habits. Sage Open. 2012;2(4):2158244012468140.

257. Aounallah-Skhiri H, Romdhane HB, Traissac P, Eymard-Duvernay S, Delpeuch F, Achour N, et al. Nutritional status of Tunisian adolescents: associated gender, environmental and socio-economic factors. Public health nutrition. 2008;11(12):1306-17.

258. Araújo MC, Ferreira DM, Pereira RA. Reliability of a semi-quantitative food frequency questionnaire designed for adolescents from the Rio de Janeiro Metropolitan Area, Brazil. Cadernos de saude publica. 2008;24(12):2775-86.

259. Arora M, Nazar GP, Gupta VK, Perry CL, Reddy KS, Stigler MH. Association of breakfast intake with obesity, dietary and physical activity behavior among urban school-aged adolescents in Delhi, India: results of a cross-sectional study. BMC Public Health. 2012;12(1):1-12.

260. Azeredo CM, de Rezende LFM, Canella DS, Claro RM, de Castro IRR, do Carmo Luiz O, et al. Dietary intake of Brazilian adolescents. Public health nutrition. 2015;18(7):1215-24.

261. CARVALHO CMRGd, NOGUEIRA AMT, TELES JBM, PAZ SMRd, SOUSA RMLd. Food consumption by adolescents enrolled in a private high school in the city of Teresina, Piauí, Brazil. Rev Nutr. 2001;14(2):85-93.

262. Estima CdCP, da Costa RS, Sichieri R, Pereira RA, da Veiga GV. Meal consumption patterns and anthropometric measurements in adolescents from a low socioeconomic neighborhood in the metropolitan area of Rio de Janeiro, Brazil. Appetite. 2009;52(3):735-9.

263. Ilesanmi OS, Ilesanmi FF, Ijarotimi IT. Determinants of fruit consumption among in-school adolescents in Ibadan, South West Nigeria. European Journal of Nutrition & Food Safety. 2014:100-9.

264. Letlape S, Mokwena K, Oguntibeju OO. Knowledge of students attending a high school in Pretoria, South Africa, on diet, nutrition and exercise. 2011.

265. López PM, Anzid K, Cherkaoui M, Baali A, Lopez SR. Nutritional status of adolescents in the context of the Moroccan nutritional transition: the role of parental education. Journal of biosocial science. 2012;44(4):481-94.

266. Monge-Rojas R, Smith-Castro V, Colón-Ramos U, Aragón MC, Herrera-Raven F. Psychosocial factors influencing the frequency of fast-food consumption among urban and rural Costa Rican adolescents. Nutrition. 2013;29(7-8):1007-12.

267. Montazerifar F, Karajibani M, Dashipour AR. Evaluation of dietary intake and food patterns of adolescent girls in Sistan and Baluchistan Province, Iran. Functional Foods in Health and Disease. 2012;2(3):62-71.

268. Naja F, Hwalla N, Itani L, Karam S, Sibai AM, Nasreddine L. A Western dietary pattern is associated with overweight and obesity in a national sample of Lebanese adolescents (13–19 years): a cross-sectional study. British Journal of Nutrition. 2015;114(11):1909-19.

269. Nurul-Fadhilah A, Teo PS, Foo LH. Validity and reproducibility of a food frequency questionnaire (FFQ) for dietary assessment in Malay adolescents in Malaysia. Asia Pacific journal of clinical nutrition. 2012;21(1):97-103.

270. Nurul-Fadhilah A, Teo PS, Huybrechts I, Foo LH. Infrequent breakfast consumption is associated with higher body adiposity and abdominal obesity in Malaysian school-aged adolescents. PloS one. 2013;8(3):e59297.

271. Peltzer K, Pengpid S. Fruits and vegetables consumption and associated factors among in-school adolescents in five Southeast Asian countries. International journal of environmental research and public health. 2012;9(10):3575-87.

272. Peltzer K, Pengpid S. Leisure time physical inactivity and sedentary behaviour and lifestyle correlates among students aged 13–15 in the association of Southeast Asian nations (ASEAN) member states, 2007–2013. International journal of environmental research and public health. 2016;13(2):217.

273. Sarkar M, Manna N, Sinha S, Sarkar S, Pradhan U. Eating habits and nutritional status among adolescent school girls: an experience from rural area of West Bengal. IOSR J Dent Med Sci. 2015;14(12):6-12.

274. Shi Z, Lien N, Kumar BN, Holmboe-Ottesen G. Socio-demographic differences in food habits and preferences of school adolescents in Jiangsu Province, China. European journal of clinical nutrition. 2005;59(12):1439-48.

275. Slater B, Philippi S, Fisberg R, Latorre M. Validation of a semi-quantitative adolescent food frequency questionnaire applied at a public school in São Paulo, Brazil. European Journal of Clinical Nutrition. 2003;57(5):629-35.

276. Xia W, Sun C, Zhang L, Zhang X, Wang J, Wang H, et al. Reproducibility and relative validity of a food frequency questionnaire developed for female adolescents in Suihua, North China. PLoS One. 2011;6(5):e19656.

277. Zhang C-X, Chen Y-M, Chen W-Q, Su Y-X, Wang C-L, Wu J-N. Food group intake among adolescents in Guangzhou city compared with the Chinese dietary guidelines. Asia Pacific journal of clinical nutrition. 2012;21(3):450-6.

278. Zanini Rd, Muniz LC, Schneider BC, Tassitano RM, do Nascimento Feitosa WM, Gonzalez-Chica DA. Daily consumption of soft drinks, sweets and fried foods among adolescents in the Northeast of Brazil. Ciencia & saude coletiva. 2013;18(12):3739.

279. Okeyo AP, Seekoe E, de Villiers A, Faber M, Nel JH, Steyn NP. Dietary practices and adolescent obesity in secondary school learners at disadvantaged schools in south africa: Urban–rural and gender differences. International Journal of Environmental Research and Public Health. 2020;17(16):1-18.

280. Neta A, Farias JC, Ferreira F, Marchioni DM. Prospective association between dietary patterns and BMI Z-score in Brazilian adolescents. Public Health Nutrition. 2021;24(13):4230-7.

281. Fatikhani DA, Setiawan A. The relationship between the level of knowledge regarding fast food and the dietary habits among adolescents in Jakarta, Indonesia. Enfermeria Clinica. 2019;29:172-5.

282. Mohammadbeigi A, Asgarian A, Ahmadli R, Fara-Shirazi SZ, Moshiri E, Ansari H, et al. Prevalence of junk food consumption, overweight/obesity and self-rated health and fitness in high school adolescent girls: A cross sectional study in a deprived area of Qom. Sri Lanka Journal of Child Health. 2019;48(3):208-14.

283. Said L, Gubbels JS, Kremers SPJ. Dietary Knowledge, Dietary Adherence, and BMI of Lebanese Adolescents and Their Parents. Nutrients. 2020;12(8).

284. Said L, Gubbels JS, Kremers SPJ. Development of Dietary Knowledge and Adherence Questionnaires for Lebanese Adolescents and Their Parents. International Journal of Environmental Research and Public Health. 2020;17(1).

285. Tunkara-Bah H, Badjan HJ, Senghore T. Dietary factors associated with being overweight and obese among school-going adolescents in Region One, The Gambia. Heliyon. 2021;7(3).

286. Giguère-Johnson M, Ward S, Ndéné Ndiaye A, Galibois I, Blaney S. Dietary intake and food behaviours of Senegalese adolescent girls. BMC Nutrition. 2021;7(1).

287. Abizari A-R, Ali Z. Dietary patterns and associated factors of schooling Ghanaian adolescents. Journal of Health, Population & Nutrition. 2019;38(1):1-10.

288. Bezerra IN, Medeiros HB, de Moura Souza A, Sichieri R. Contribution of away-from-home food to the energy and nutrient intake among Brazilian adolescents. Public Health Nutrition. 2021;24(11):3371-8.

289. Agofure O, Odjimogho S, Okandeji-Barry O, Moses V. Dietary pattern and nutritional status of female adolescents in Amai Secondary School, Delta State, Nigeria. The Pan African Medical Journal. 2021;38.

290. Thorne-Lyman AL, Shaikh S, Mehra S, Wu LSF, Ali H, Alland K, et al. Dietary patterns of &gt;30,000 adolescents 9–15 years of age in rural Bangladesh. Annals of the New York Academy of Sciences2020. p. 3-15.

291. Teixeira MT, Vitorino RS, da Silva JH, Raposo LM, Aquino LAd, Ribas SA. Eating habits of children and adolescents during the COVID‐19 pandemic: The impact of social isolation. Journal of Human Nutrition & Dietetics. 2021;34(4):670-8.

292. Korkalo L, Erkkola M, Fidalgo L, Nevalainen J, Mutanen M. Food photographs in portion size estimation among adolescent Mozambican girls. Public Health Nutrition. 2013;16(9):1558-64.
